# Association between climate change awareness-related psychological distress and mental health in people with psychiatric diagnoses or subclinical symptoms: a scoping review

**DOI:** 10.1192/j.eurpsy.2026.10169

**Published:** 2026-02-18

**Authors:** Hendrik Peuskens, Johan Detraux, Kirsten Catthoor, Kris Van den Broeck, Victor-Emiel Bellens, Thomas Vandendriessche, Chayenne Van Meel, Marc De Hert, Jurjen J. Luykx, Maarten Van Den Bossche, Manuel Morrens

**Affiliations:** 1 Alexianen Zorggroep Tienen, Psychiatric Clinic, Tienen, Belgium.; 2 University Psychiatric Center KU Leuven, Kortenberg, Belgium.; 3 Research Group Psychiatry, KU Leuven, Leuven, Belgium.; 4 Ziekenhuis Netwerk Antwerpen (ZNA), Antwerp, Belgium.; 5 Flemish Psychiatric Association, Kortenberg, Belgium.; 6 Collaborative Antwerp Psychiatric Research Institute (CAPRI), University of Antwerp, Antwerp, Belgium.; 7 Family Medicine and Population Health (FAMPOP), Faculty of Medicine and Health Sciences, University of Antwerp, Antwerp, Belgium.; 8 Faculty of Medicine and Health Sciences, Ghent University, Ghent, Belgium.; 9 KU Leuven Libraries - 2Bergen – Learning Centre Désiré Collen, 3000 Leuven, Belgium.; 10 Department of Neurosciences, Research Group Psychiatry, Center for Clinical Psychiatry, KU Leuven, Leuven, Belgium.; 11 Antwerp Health Law and Ethics Chair, AHLEC University Antwerpen, Antwerp, Belgium.; 12 Department of Psychiatry, Amsterdam University Medical Center, Amsterdam, The Netherlands.; 13 GGZ inGeest Mental Health Care, Amsterdam, The Netherlands.; 14 Neuroscience Mood, Anxiety, Psychosis, Stress & Sleep Research Program, Amsterdam University Medical Center, Amsterdam, The Netherlands.; 15 Department of Psychiatry and Neuropsychology, School for Mental Health and Neuroscience, Maastricht University Medical Center, Maastricht, The Netherlands.; 16 Public Health Mental Health Research Program, Amsterdam University Medical Center, Amsterdam, The Netherlands.; 17 Geriatric Psychiatry, University Psychiatric Center KU Leuven, Leuven, Belgium.; 18 Neuropsychiatry, Research Group Psychiatry, Department of Neurosciences, Leuven Brain Institute, KU Leuven, Leuven, Belgium.; 19 Scientific Initiative of Neuropsychiatric and Psychopharmacological Studies (SINAPS), University Psychiatric Centre Duffel, Duffel, Belgium.

**Keywords:** climate awareness, climate anxiety, mental health, psychological distress, mental disorder

## Abstract

**Background:**

The awareness of climate change as a global environmental threat through media consumption and/or social interaction can have a psychological impact on people’s mental health. However, little is known about the association between climate change awareness-related psychological distress (CCARPD) and mental health in people with psychiatric diagnoses or subclinical symptoms.

**Methods:**

A comprehensive and systematic literature search of the PubMed, Embase, Web of Science Core Collection, Scopus, and CENTRAL electronic databases (from inception to February 2025) was conducted, without language restriction, for articles assessing the association between CCARPD and the mental well-being of people in the general and psychiatric populations.

**Results:**

Twenty-eight thousand forty-seven reports were retrieved. Of these, 67 met the inclusion criteria (64 general and 3 psychiatric population studies). The overall correlation between CCARPD and mental health measurements (ranging from subclinical symptoms to clinical diagnoses of depression, anxiety, or stress) was positive and of weak-to-moderate strength. Nevertheless, higher psychological distress due to the awareness of climate change was found in those having more severe mental health problems.

**Conclusions:**

Although most studies have found small-to-moderate correlations between CCARPD and mental health measurements, it can be distressing and damaging for those with more severe mental health problems. As CCARPD will increase globally as the climate crisis unfolds in the coming decades while the understanding of the connections between CCARPD and mental well-being is still at an early stage of development, more research will be of utmost relevance, particularly in psychiatric populations.

## Introduction

Anthropogenic climate changes have been identified by the United Nations and the World Health Organization as “the defining issue of our time” and “the greatest threat to global health in the 21st century” [1–5]. Although most of the attention has been placed on physical health impacts of climate changes, mental health challenges are also expected to increase [6–10].

Climate change can affect mental health *directly* due to acute events (e.g., the development of a post-traumatic stress disorder [PTSD] after climate-related disaster exposure), or *indirectly* through slow-moving consequences of climate change on landscape or agriculture (e.g., the development of psychological distress among indigenous people due to drought, the rising of sea levels, or the loss of biodiversity). However, even without experiencing the direct or indirect effects of climate change, simply the awareness or perception of climate change as a global environmental threat through media consumption (including newspapers, scientific articles, social media, radio, television, and the internet) and/or social interactions also can have a psychological impact on people’s mental health [8, 9, 11–28].

Public awareness of the effects of climate change is rising due to growing media coverage of climate change-related consequences [8, 29]. Since 2007, media reports on climate change have increased by almost 80% [30]. Moreover, media dissemination of information about climate change frequently contains items highlighting the likely negative, even catastrophic consequences of these changes [27]. This explains why large-scale survey studies have shown that the majority of people believe that climate change is a global emergency [31, 32].

Although people with preexisting (severe) mental illness are particularly vulnerable to the direct and indirect impacts of climate change [13, 16, 33–35], they may also be more vulnerable to increased psychological distress as a consequence of thinking about the future threat that climate change poses [27, 36–41]. Constant media exposure to these sources of information and images may engender anxiety, depressive, or stressful symptoms [42], possibly leading to an exacerbation of their disease [17, 23, 43, 44]. However, little is known about the relationship between climate change awareness-related psychological distress (CCARPD) and the mental health of people with preexisting mental illness. Therefore, the objective of this study was to conduct a scoping review to systematically map the research done on the association between CCARPD and the mental health of people with preexisting lifetime or current mental illness, as well as to identify the existing gaps in this research. Due to the expected small number of studies available in this research field, we decided to include also studies using samples of people with mental health vulnerabilities from the general population (i.e., people with subclinical symptoms or people having previously received treatment or support for mental health problems/diagnoses undermining daily functioning).

## Methods

This scoping review was conducted according to the 2018 Preferred Reporting Items for Systematic reviews and Meta-Analyses extension for Scoping Reviews (PRISMA-ScR) guidelines [45], and the 2020 scoping reviews chapter in the JBI Manual for Evidence Synthesis [46], which is congruent with the PRISMA-ScR checklist. The protocol was registered with the Open Science Framework initiative (https://osf.io/t59mg/) on May 5, 2023.

### Search strategy

A comprehensive and systematic literature search in PubMed (via NCBI, including MEDLINE), Embase (Embase.com), Web of Science Core Collection (SCI-EXPANDED, SSCI, AHCI, CPCI-S, CPCI-SSH, ESCI), Scopus (Scopus.com), and CENTRAL (via Cochrane Library) electronic databases (from inception to February 2025) was conducted on April 13, 2023 (with updates on July 1, 2024 and February 27, 2025). The search was performed without language restriction to identify records assessing the association between CCARPD and the mental well-being of people with psychiatric diagnoses or subclinical symptoms in psychiatric and general populations. Four of the authors, of whom two are experienced biomedical information specialists, closely worked together to construct search strings for the different databases. Full search strings are available in the Supplementary Material. Duplicates of the original search were removed, using EndNote 20 (based on the instructions provided by Leeds University Library).^1^ After removing duplicates, titles and abstracts were screened, using Rayyan QCRI. Articles that were deemed potentially relevant according to the selection criteria were included. Any doubts were solved by consensus or by decision of a second and/or third reviewer. We also attempted to identify additional studies through a process of backward citation searching, using the references of identified relevant reports on CCARPD and mental health [47].

### Selection criteria

All inclusion and exclusion criteria are presented in Table 1. We included studies exploring the relationship between CCARPD (anxiety, depression, and/or stress) and the mental health of people with preexisting or probable mental illness. CCARPD operates along a single continuum from mild concern to severe psychological distress [49]. As clinicians, we decided to focus on the more severe forms of CCARPD, as these are especially relevant as indicators of poor mental health [50–54]. Environmental worry or concern, a cognitive phenomenon with affective repercussions but without interfering with one’s daily functioning [55, 56], for example, is often considered to be a less severe form of CCARPD [50, 55, 57]. Therefore, we only included studies using at least some functional impairment measure [48], meaning that the CCARPD had to interfere with a person’s ability to work, study or socialize, leading to a reduction in quality of life.

**Table 1.** Inclusion and exclusion criteria of studies

Parameter	Inclusion criteria	Exclusion criteria
Category of research information	Published, peer-reviewed studies/records.	Studies that were not peer-reviewed or published (grey literature)
Study design	Population-based observational studies (including case-control, prospective and retrospective cohort studies, or cross-sectional studies), survey studies, or naturalistic studies. Quantitative and mixed methods studies.	Case reports, qualitative studies, studies on education or therapeutic strategies for CCARPD, viewpoints, recommendations, editorials, preprints, dissertations, conference abstracts/papers, books/book sections, or study protocols were excluded.
Population	*Psychiatric population studies*, consisting of children, adolescents, adults, or elderly with a clinically confirmed preexisting lifetime or current psychiatric disorder with a strong link to anxiety, depression and/or stress. Diagnoses had to be made on the basis of a widely-accepted standardized disease coding system, such as the Diagnostic and Statistical Manual of Mental Disorders (DSM) or the International Classification of Diseases (ICD), a structural diagnostic interview based on these coding systems, such as the SCID (Structural Diagnostic Interview for DSM-IV disorders), or medical records. *General population studies*, consisting of children, adolescents, adults, or elderly with mental health vulnerabilities, where screening tools with validated cut-off scores that aid in identifying individuals at risk for a clinical diagnosis were used (such as the Center for Epidemiological Studies-Depression [CES-D] [a self-report measure of depression], the Patient Health Questionnaire [PHQ] [a scale assessing most of the criteria for major depressive disorder in the DSM], or the General Anxiety Disorder [GAD] scale [a scale assessing generalized anxiety symptoms]), or where the investigators relied on medical records.	Studies that did not include patients with preexisting or probable mental illness.
Exposure	Studies reporting on climate change awareness-related psychological distress by using empirically validated scales, such as **the Climate Change Anxiety Scale (CCAS)**. The **Climate Change Anxiety Scale (CCAS)** *is a 22-item scale measuring the emotional response to climate change. The questions measure four variables including cognitive-emotional impairment, functional impairment, personal experience of climate change, and behavioral engagement.*	Studies examining mental health disorders or CCARPD due to extreme weather events or indirect consequences of climate changes (e.g., studies examining the impact of climate change on the psychological well-being of indigenous people who maintain close connections to the environment). Studies examining the impact of less severe forms of CCARPD (thus without functional impairment, such as environmental concern or worry). Studies using non-validated assessments of mental health or CCARPD or single-item measures of CCARPD (as these often fail to capture the multifaceted nature of CCARPD, risking oversimplification and inconsistent data due to cultural and contextual variability) [48], studies focusing solely on related concepts, such as solastalgia, eco-guilt, or eco-grief.
Outcome	Studies reporting on the mental health of people with preexisting psychiatric diagnoses or subclinical symptoms by using empirically validated scales, such as the **Generalized Anxiety Disorder (GAD)** scale, the **Beck Depression Inventory (BDI)-II**, the **Patient Health Questionnaire (PHQ)-8**, the **Depression Anxiety Stress Scales (DASS)-42**, or the **Hospital Anxiety and Depression (HAD)-14** scale. *The **Generalized Anxiety Disorder (GAD)-7,** one of the most commonly used tools in epidemiological anxiety research, is a 7-item scale for assessing generalized anxiety symptoms over a 2-week period. Participants rate 7 statements about anxiety, preceded by the sentence “Over the last two weeks, how often have you been bothered by the following problem?*”, *on a 4-point Likert-type scale (0: “never” to 3: “almost every day”). Scores of 10 or greater are indicative of probable GAD.* *The **Beck Depression Inventory (BDI)-II** is a 21-item instrument designed to measure both the presence and severity of depressive symptoms over a 2-week period. Each item consists of a group of four statements measuring the symptoms of depression that range in intensity, with each item scored on a scale value of 0–3. The total score is interpreted according to different levels of depression severity: minimal (0–13), mild (14–19), moderate (20–28), and severe (29–63).* *The **Patient Health Questionnaire (PHQ)-8** assesses eight of the nine criteria for MDD in the DSM, excluding symptoms of suicidality. Scores of 10 or above are considered indicative of a probable MDD diagnosis.* *The **Depression Anxiety Stress Scales (DASS)-42**, a widely used self-report questionnaire with 42 items, assesses the three following dimensions: depression, anxiety, and stress. Final scores are interpreted using the following ranges: Depression: normal (0–13), mild (14–20), moderate (21–27), severe (28–34), and extremely severe (35+). Anxiety: normal (0–9), mild (10–13), moderate (14–20), severe (21–27), and extremely severe (28+). Stress: normal (0–14), mild (15–18), moderate (19–25), severe (26–33), and extremely severe (34+)* *The **Hospital Anxiety and Depression (HAD)-14** is a 14-item scale rated from 0 to 3. Seven questions are related to the anxiety dimension (HAD-A) and seven others to the depressive dimension (HAD-D). Doubtful symptomatology if one has a score between 8 and 10; definite symptomatology if the score is 11 or more.*	Studies using non-validated assessments of mental health.
Comparator	A comparator or control group was not necessary.	N/A

Abbreviations: CCARPD, Climate Change Awareness-Related Psychological Distress; MDD, Major Depressive Disorder; N/A, Not Applicable.

### Data extraction

Data were extracted and mapped descriptively by JD, using a data extraction form, one for studies done within the general population, and one for studies within the clinical population. This extraction form included the following information:

- author(s);
- country (where the study was conducted);
- study design;
- patient characteristics (sample size, female gender, mean age, specific information on clinical symptoms, or previous psychological support);
- information on screening tools and on outcomes collected as an exposure or mental health outcome;
- outcomes/key findings that relate to the scoping review questions.

As a meta-analysis was not feasible, due to the expected high methodological heterogeneity between studies, descriptive statistics were used to summarize study and patient characteristics.

## Results

### Search strategy

**Figure 1.**
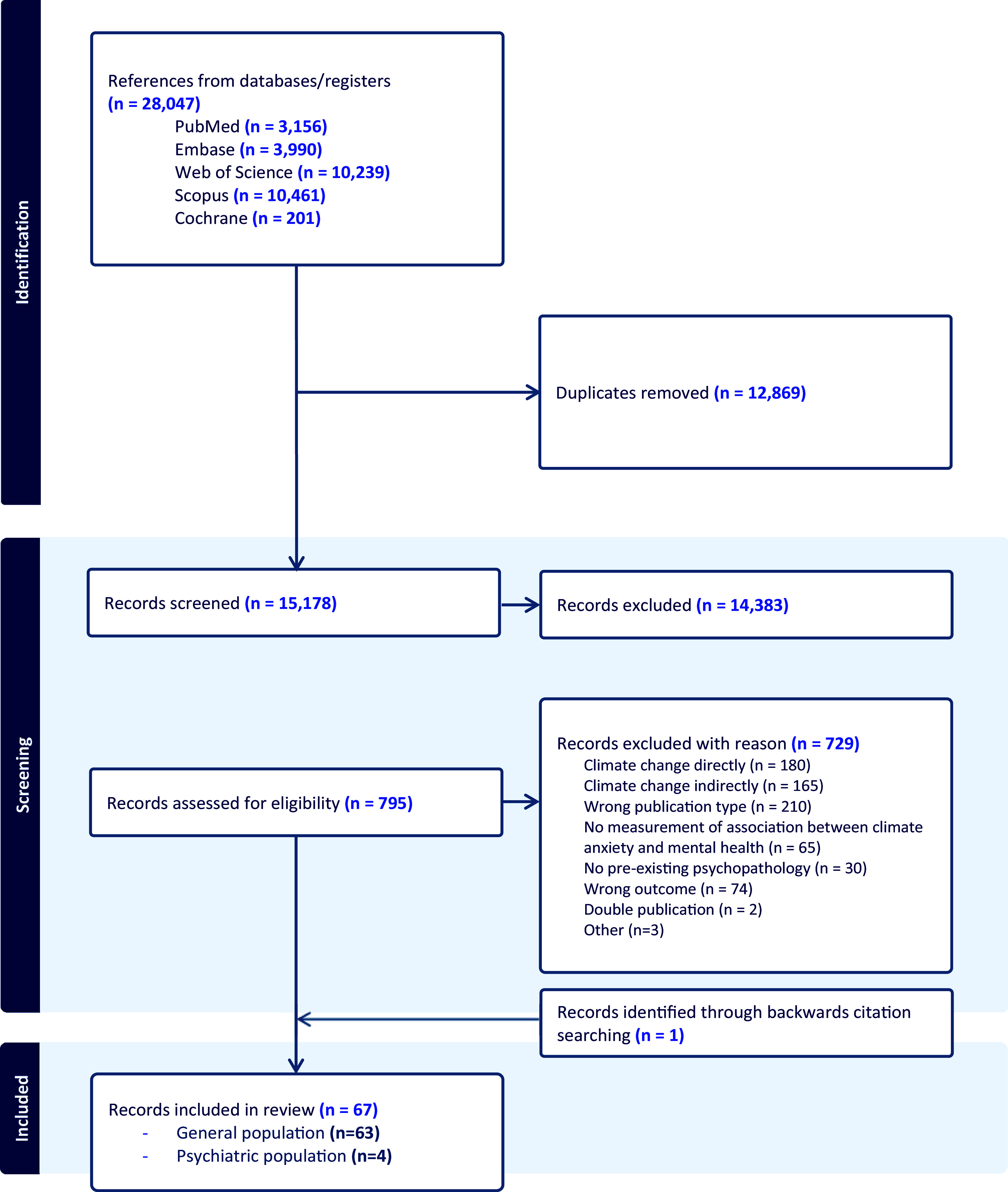
PRISMA flowchart.

The search in PubMed (n = 3,156), Embase (n = 3,990), Web of Science Core Collection (n = 10,239), Scopus (n = 10,461), and Cochrane (n = 201) databases yielded a total of 28,047 reports. From these, 12,869 duplicates were removed. Overall, 795 references of published records were selected as potentially eligible, of which 67 records [6, 18, 27, 43, 50–55, 58–114] met the inclusion criteria. One record [61] was identified through backward citation searching. The results of the study selection are shown in the PRISMA flowchart (see Figure 1).

### Study and patient characteristics

Sixty-seven records were included in this scoping review (see Tables 2–4). Sixty-four studies were conducted in the general population; three concerned psychiatric population studies. Most general population studies had a cross-sectional design (n = 61) (of which one [77] also included MRI data, two [104, 105] were based on the same patient population, and three [18, 96, 110] had a mixed methods design (presenting quantitative and qualitative data). Veijonaho et al. [81], Whitmarsh et al. [54], and Chan et al. [50] were longitudinal studies. Two of the psychiatric population records were cross-sectional studies [113, 114], one [27] a naturalistic study. Mean age of the participants for the general and psychiatric population studies (N = 47,687) was 32 years (range: 14–55 years). Sample sizes ranged from 42 to 3,091 participants. All studies, except the Yesildere Saglam and Sahin [62], Chan et al. [67], Weiß et al. [70], Hogg et al. [75], Veijonaho et al. [81], Lukacs et al. [98], Feather and Williams [103], Hajek and König [104, 105], Clayton and Karazsia [112], and Jones et al. [27] studies, included more female participants (mean: 63,8%). Forty percent of the studies were conducted in Europe (Germany, n = 12; France, n = 6; Italy, n = 3; Poland, n = 4; Slovenia, n = 1; Finland, n = 1). The other studies were done in Australia/New Zealand (n = 9), the United States (n = 5), Turkey (n = 6), Canada (n = 4), the United Kingdom (n = 3), China (n = 2), Lebanon (n = 2), Libya (n = 1), South Africa (n = 1), or multiple countries (n = 7). Most studies used specifically designed scales to measure climate change anxiety, which were in most cases the Climate Change Anxiety Scale (CCAS) (n = 44) and the Hogg Eco-Anxiety Scale (n = 15). Three studies used the Climate Distress Scale, two the Eco-Anxiety Questionnaire and one the Climate Change Distress and Impairment Scale. Nearly three-fourth of the non-clinical population studies (n = 33) measured two or more categories of symptoms (i.e., depression, anxiety, and/or stress), using one or more of the above-mentioned scales.

**Table 2.** Study and patient characteristics of studies in general population individuals with subclinical symptoms or mental diagnoses [6, 18, 43, 50–55, 58–112]

Author(s)	Country	Study design	N	Female (%)	Mean age (SD), range (years)	Specific information on clinical symptoms or previous psychological support	Scales
Lau et al. [58]	China	CSD	1,567	72	27.8 (11.6), 18–30 (74.4%), 30< (25.6%)		CCAS PHQ-4 WHO-5
Avan and Vural [59]	Turkey	CSD	883	62.5	16.1 (1.1), 14-17		CCAS QDIA-SR Bergen Insomnia Scale
Li et al. [60]	China	CSD	653	53.8	32.6 (7.4), NR		CCAS GAD-7
Cameron and Kagee [61]	South Africa	CSD	343	NR	NR (NR), 18–23 (92.7%)		CCAS GAD-7 PHQ-9
Yesildere Saglam and Sahin [62]	Turkey	CSD	456	100	26.9 (7.0), 18≤	44.7% (n = 204) had PMS; (n = 252) had no PMS	HEAS PMSS
Cosh et al. [63]	Australia	CSD	877	72.2	21.1 (2.6), 16–25		CCAS DASS-21
Shepherd et al. [64]	France	CSD	442	82.1	32.5 (12.5), 18–77		CCAS DASS-42 PSWQ
Wagner and Witthöft [65]	Germany	CSD	415	67	32.6 (12.4), 16–83		CCAS PHQ-4
Coates et al. [66]	Australia	CSD	236	64.8	27.8 (12.2), 18–84		CCAS DASS-21 (7 anxiety items)
Chan et al. [50]	US	Longitudinal	682	57.7	46.2 (7.1), NR		CCAS GAD-7
Chan et al. [67]	US	CSD	1,004	52	NR (NR), 18–65+		CCAS GAD-7 PHQ-8
	China	CSD	1,009	49.3	NR (NR), 18–65+		CCAS GAD-7 PHQ-8
Becht et al. [68]	The Netherlands/Colombia	CSD	537	51.2	17.7 (NR), 16–18		CCAS GAD-7
Larionow et al. [69]	Poland	CSD	634	81.4	28.1 (10.7), 18–67		HEAS PHQ-4 WHO-5
Weiß et al. [70]	Germany	CSD	306	49	42.9 (13.0), 18–75		CCAS GAD-7 PHQ-2
Ali et al. [71]	Libya	CSD	1,619	85.7	22.1 (3.7), 18–54		HEAS DASS-8
Brailovskaia and Teismann [72]	Germany	CSD	323	68.4	26.1 (8.4), 18–63		CCDIS DASS-21 SIBS SDES
Pitron et al. [73]	Germany	CSD	421	67	32.7 (12.4), 16–83		CCAS PHQ-4 SSS-8 MHW-12
Tucholska et al. [74]	Poland	CSD	333	68.8	29.1 (11.2), 18–80		CCAS BFI-15
Hogg et al. [75]	UK, US	CSD	1,009	UK: 50.1 US: 49.8	UK (n = 501): 41.9 (12.1), 18–77 US (n = 508): 36.3 (12.9), 18–82		HEAS PSWQ
Fekih-Romdhane et al. [76]	Lebanon	CSD	763	63.4	28.6 (11.1), NR		CCAS DASS-8
Carlson et al. [77]	US	CSD-MRI	42	73.8	20.7 (2.5), NR		CCAS DASS-42
Zeier and Wessa [78]	Germany	CSD	871	56.4	49.3 (15.3), NR		CCAS EAQ-22 PHQ-4
Fekih-Romdhane et al. [79]	Lebanon	CSD	596	63.9	23.5 (4.1), 18–35		CCAS SDA PQ-B
Çimşir et al. [80]	Turkey	CSD	445	64.3	29.8 (NR), 18–65		HEAS BSI
Veijonaho et al. [81]	Finland	Longitudinal	3,002	47	NR (NR), 11–15		CCAS (adapted 8-item version) DEPS-10
Orrù and Mannarini [82]	Italy	CSD	343	73.5	29.9 (13.4), NR		HEAS DERS-SF
Lykins et al. [83]	Australia	CSD	103	100	30.8 (4.9), 19-45		CCAS EPDS
Hogg et al. [84]	Australia	CSD	530	63.2	39.5 (16.5), 18–86		HEAS GAD-7 DASS-21 (depression subscale)
Wullenkord et al. [85]	Germany	CSD	2,052	51	44.7 (14.4), 18–82		HEAS Cumulative stressors (5 items)
Parmentier et al. [86]	France	CSD	431	71.7	37.6 (14.6), 18–78		CCAS STAI-Y
Jalin et al. [87]	France	CSD	522	51.7	28.5 (12.8), 18–76		EMEA GAD-7 PHQ-9
Micoulaud-Franchi et al. [88]	France	CSD	1,004	54.1	43.5 (13.4), 19–66	31.1% and 14.9% of the participants had current clinically significant anxiety and depressive symptoms, respectively	EAQ-22 HAD
Er et al. [89]	Turkey	CSD	609	84.2	NR (NR), 18–20 (60.8%) and ≤21 (39.2%)		HEAS DASS-21
Nezlek and Cypryańska [90]	Poland	CSD	1,133	53	46.7 (15.5), ≤18		CDS CES-D-8 GAD-4
Plohl et al. [91]	Slovenia	CSD	442	75.8	21.6 (1.7), 18–24		CCAS DASS-21 BFI-15 (neuroticism measured by 6-item negative emotionality subscale) MHC-SF (emotional, social, and psychological well-being subscales)
Asgarizadeh et al. [53]	Canada/US	CSD	323	64.7	54.6 (16.7), 19–87		CCAS GAD-7
Patrick et al. [92]	Australia	CSD	2,359	56.5	52.7 (19.96), 18–75+		CCAS Pre-traumatic stress scale
Thomson and Roach [93]	Canada	CSD	327	72.2	24.3 (9.2), 18–68 (81% of the participants were under 30 years of age)		CCAS DASS-21
Türkarslan et al. [6]	Turkey	CSD	605	69.9	26.5 (8.3), 18–68		HEAS DASS-21
Ediz and Yanik [94]	Turkey	CSD	306	75.2	NR (NR), 15–24	29.1% of the participants had previous psychological support	CCAS BHS
Wu et al. [51]	Canada	CSD	2,306	NR	16.3 (0.5), 15–18		CCAS-S PHQ-8 GAD-2
Hepp et al. [52]	Various (most participants from UK, US, Canada, and South Africa)	CSD	374 (study 3)	50.3	30.7 (9.2), 18–59	24.87% of the participants had a mental health diagnosis.	CCDIS CCAS BDI-II GAD-7 NEO-FFI (neuroticism subscale) PANAS-X
Mathers-Jones and Todd [95]	Australia	CSD	96	70.8	20.86 (3.4), 18–31		HEAS DASS-21
Vercammen et al. [96]	UK	MMS	539	60	NR (NR), 16–24		CDS (adapted 8-item version) Respondents were asked whether their thoughts and feelings about climate change ever interfered with their well-being or day-to-day functioning PHQ-9 GAD-7 PSS Respondents were asked whether they had ever received a diagnosis of, or treatment for, a mental health condition, and/or whether they had ever experienced mental ill health (regardless of specific diagnostic criteria or treatment)
Heinzel et al. [97]	Germany	CSD	486	73.5	29.4 (10.6), 18–73		HEAS DASS-21
Lutz et al. [55]	Canada	CSD	308	78.6	20.2 (4), 17–47		CCAS-21 DASS SPANE
Lukacs et al. [98]	Canada	CSD	1,553	46.4	NR (NR), 16–65+		CCAS K-6
Mathé et al. [99]	France	CSD	275	66.2	31.2 (13.9), 18–80		HEAS CCAS DASS-21 STAI-Y
Innocenti et al. [100]	Italy	CSD	150	52.7	34.1 (11.1), 19–76		CCAS HEAS DASS-21
Schwartz et al. [18]	US	CSD (MMS)	284	78.9	23.2 (3.9), 18–35		CCAS (13 statements concerning cognitive-emotional and functional impairment) PHQ-8 GAD-7
Heeren et al. [101]	Belgium, France, Switzerland	CSD	874	51.4	38.4 (14.1), 18–81		CCAS PSWQ
Schwaab et al. [102]	Germany	CSD	203	58	25.2 (3.7), NR		PHQ-9/PHQ-9-C GAD-7/GAD-7-C PTSS-10/PTSS-10-C PSQ-20/PSQ-20-C
Whitmarsh et al. [54]	UK	Longitudinal	1,338 (T1) and 891 (T2)^a^	53	47.1 (NR), 18–85		CCAS (13 statements concerning cognitive-emotional and functional impairment) GAD-7
Feather and Williams [103]	Australia/New Zealand	CSD	771	48	33 (11.9), 18–81		Modified CCAS (removed 6 items) PHQ-4
Hajek and König [104, 105]	Germany	CSD	3,091	49.5	46.5 (15.3), 18–74		CCAS PHQ-9 GAD-7 De Jong Gierveld loneliness scale PSIS
Mouguiama-Daouda et al. [106]	Several French-speaking countries (mainly France,89%)	CSD	305	72.1	30.8 (11.3), 17–70		CCAS GAD-7 BDI-II
Larionow et al. [107]	Poland	CSD	603	57.1	25.3 (9.6), 18–70		CCAS PHQ-4
Cruz and High [108]	US	CSD	513	61	52.2 (18.5), NR		CCAS CES-D-10 STAIT-5
Lawrance et al. [43]	UK	CSD	530	63	21.0 (2.5), 16–24	12% reported a previous experience of a mental health disorder; 3% had recently been diagnosed or were receiving treatment for the first time; 16% were managing an ongoing mental health issue.	CDS (adapted 8-item version) Respondents were asked whether their thoughts and feelings about climate change ever interfered with their well-being or day-to-day functioning GAD-7 PHQ-9 PSS-4
Wullenkord et al. [109]	Germany	CSD	1,011	51.1	43.9 (14), 18–69		CCAS PHQ-4
Hogg et al. [110]	Australia/New Zealand	MMS	Australian study 334 New Zealand study *Sample 1* 365 *Sample 2* 370	Australian study 59 New Zealand study *Sample 1* 80 *Sample 2* 70	Australian study 22.2 (6.7), 17–65 New Zealand study *Sample 1* 19.9 (3.6), NR *Sample 2* 19.4 (2.9), NR		HEAS GAD-7 DASS-21
Innocenti et al. [111]	Italy	CSD	130	67.4	32.8, (11.7), 19–76		CCAS GAD-7 K-10
Clayton and Karazsia [112]	US	CSD	197	40.6	NR (NR), about 50% between 25 and 34, but ranged from the category 18–24 to three people who were 75 or older		CCAS PHQ-4

Abbreviations: BDI-II, Beck Depression Inventory-II; BFI-10, Short 10-item Big Five Inventory; BFI-15, Short 15-item Big Five Inventory; BHS, Beck Hopelessness Scale; BSI, Brief Symptom Inventory; CCA, Climate Change Anxiety; CCARS, Climate Change Awareness-Related Distress; CCAS, Climate Change Anxiety Scale; CCAS-S, Climate Change Anxiety Scale Short-Form (abbreviated 4-item version of the CCAS); CCDIS, Climate Change Distress and Impairment Scale; CDS, Climate Distress Scale; CES-D-10, Center for Epidemiologic Studies Depression Scale, 10-item version; CES-D-8, Center for Epidemiologic Studies Depression Scale, 8-item version; CSD, Cross-Sectional Design; DASS-8, Depression Anxiety Stress Scale, 8-item version; DASS-21, Depression Anxiety Stress Scale, 21-item version; DASS-42, Depression Anxiety Stress Scale, 42-item version; DEPS-10, The Depression Scale, 10-item version; DERS-SF, Difficulties in Emotion Regulation Scale – Short Form; DT, Distress Thermometer; EAQ-22, Eco-Anxiety Questionnaire; EID, Environmental Identity Scale; EMEA, Eco-Anxiety Measurement Scale; EPDS, Edinburgh Postnatal Depression Scale; GAD, General Anxiety Disorder; GAD-9, General Anxiety Disorder scale, 9-item version; GAD-7, General Anxiety Disorder scale, 7-item version; GAD-7-C, The climate change specific General Anxiety Disorder scale, 7-item version; GAD-2, General Anxiety Disorder scale, 2-item version; GAD-4, General Anxiety Disorder scale, 4-item version; HAD, Hospital Anxiety and Depression Scale; HEAS, Hogg Eco-Anxiety Scale; K-10, Kessler Psychological Distress Scale, 10-item version; K-6, Kessler Psychological Distress Scale, 6-item version; MDD, Major Depressive Disorder; MHC-SF, Mental Health Continuum-Short Form; MHW-12, Modern Health Worries scale (12 questions); MRI, Magnetic Resonance Imaging; MMS, Mixed-Methods Study; NEO-FFI, NEO Five-Factor Inventory; NR, Not Reported; PANAS-X, Positive and Negative Affect Schedule- expanded form; PHQ-4, Patient Health Questionnaire for depression and anxiety, 4-item version; PHQ-8, Patient Health Questionnaire for depression, 8-item version; PHQ-9, Patient Health Questionnaire for depression, 9-item version; PHQ-9-C, The climate change specific Patient Health Questionnaire for depression, 9-item version; PMS, Premenstrual Syndrome; PMSS, Premenstrual Syndrome Scale; PQ-B, Prodromal Questionnaire-Brief; PSIS, Perceived Social Isolation Scale; PSS-4, Perceived Stress Scale, 4-item version; PSWQ, Penn State Worry Questionnaire; PSQ-20, Perceived Stress Questionnaire, 20-item version; PSQ-20-C, the climate change specific Perceived Stress Questionnaire, 20-item version; PTSD-8, Post-Traumatic Stress Disorder, 8-item questionnaire; PTSS-10, Posttraumatic Stress Scale, 10-item version; PTSS-10-C, The climate change specific Posttraumatic Stress Scale, 10-item version; QDIA-SR, Quick Depression Inventory for Adolescents-Self-Report Form; SDA, Scale of Death Anxiety; SDES, Short Defeat and Entrapment Scale; SDQ, Strengths and Difficulties Questionnaire; SIBS, Suicide Ideation and Behavior Scale; SPANE, Scale of Positive and Negative Experiences; SSS-8, Somatic Symptoms Scale (8 categories of somatic symptoms); STAIT-5, State-Trait Anxiety Inventory Scale-5 questionnaire; STAI-Y, Spielberger Trait Anxiety Scale-Form Y; UK, United Kingdom; US, United States; WHO-5, World Health Organization-Five Well-Being Index.

a Since the initial data were collected during the COVID-19 pandemic, potentially affecting climate anxiety levels, the sample was recontacted in late May 2022 to complete the climate anxiety measure a second time; 891 respondents completed the measure. Three percent of the sample had experienced flood damage to their home or garden in the last 5 years.

**Table 3.** Results of studies in general population individuals with subclinical symptoms or mental diagnoses [6, 18, 43, 50–55, 58–112]

Author(s)	Exposure	Mental health outcomes	Results
Lau et al. [58]	CCARPD	Depression and anxiety symptoms, and mental well-being	CCAS had a statistical significant, albeit weak, positive relationship with depression and anxiety symptoms (r = 0.21, p < 0.01) and a negative relationship with the WHO-5 (r = −0.073, p < 0.01).
Avan and Vural [59]	CCARPD	Depression symptoms and insomnia	An increase in CCARPD is associated with higher levels of depression (r = 0.10, p < 0.01) and insomnia (r = 0.19, p < 0.01) in adolescents (39.6% and 15.4% of the sample had severe and moderate depression, respectively).
Li et al. [60]	Green space exposure	CCARPD and generalized anxiety symptoms	Generalized anxiety disorder was positively correlated with both cognitive-emotional impairment (r = 0.52, p < 0.05) and functional impairment (r = 0.59, p < 0.05) related to climate change anxiety.
Cameron and Kagee [61]	Depression and anxiety symptoms	CCARPD	CCARPD is statistically significantly (p < 0.001) correlated with GAD-7 (r = 0.38) and PHQ-9 (r = 0.28). When considered together, F(2, 340) = 28.43, p < 0.001, general anxiety and depression predicted 14.3% of the variance in CCARPD.
Yesildere Saglam and Sahin [62]	CCARPD	Premenstrual symptoms (e.g., depressive affect, anxiety, depressive thoughts)	Women with clinically relevant PMS had higher HEAS scores (mean: 30.6), compared to women without PMS (mean: 24.6) (p < 0.001). HEAS scores were significant predictors of PMS (OR = 0.828, 95% CI: 0.791–0.867, p < 0.001).
Cosh et al. [63]	CCARPD	Depression, anxiety, and stress symptoms	A cut-off CCAS score of 21 is proposed for detecting mild to moderate cases of CCARPD and a cut-off CCAS score of 23 to detect severe cases of CCARPD (33% of the sample reported extremely severe anxiety symptoms, while 9% and 20% had extremely severe stress and depression, respectively).
Shepherd et al. [64]	CCARPD	Depression, anxiety, and stress symptoms	The CCAS showed moderate positive correlations with measures of general psychopathology (DASS) and general worry (PSWQ) with correlations ranging from 0.31 to 0.37 (all p < 0.001).
Wagner and Witthöft [65]	CCARPD	Depression and anxiety symptoms	There was a positive correlation between CCARPD and depression/anxiety (r = 0.44, p < 0.001).
Coates et al. [66]	CCARPD	Anxiety symptoms	A significant positive relationship between climate anxiety and participants’ general levels of anxiety was found (r = 0.540, p < 0.001).
Chan et al. [50]	CCARPD	Anxiety symptoms	No direct association between generalized anxiety and cognitive-emotional or functional impairment over time was found. Time 1 cognitive-emotional impairment (b = −0.08, SE = 0.07, p = 0.257, 95% CI = [− 0.21, 0.06], standardized beta = −0.05) and Time 1 functional impairment (b = −0.04, SE = 0.08, p = 0.570, 95% CI = [−0.19, 0.11], standardized beta = −0.03) were unrelated to the change in Time 2 generalized anxiety.
Chan et al. [67]	CCARPD	Depression and anxiety symptoms	Association between CCARPD and generalized anxiety (r = 0.35) and depression (r = 0.38).
	CCARPD	Depression and anxiety symptoms	Association between CCARPD and generalized anxiety (r = 0.56) and depression (r = 0.55).
Becht et al. [68]	CCARPD	Anxiety symptoms	A moderate correlation (r = 0.31, p < 0.001) was observed between CCAS and GAD-7 scores.
Larionow et al. [69]	CCARPD	Depression and anxiety symptoms, and mental well-being	All HEAS subscale scores were statistically significantly and positively associated with anxiety and depression (correlations between r = 0.14 to r = 0.56, all p < 0.001). In contrast, all HEAS subscale scores were statistically significantly and negatively associated with well-being (correlations between r = −0.09, p < 0.05 to r = −0.28, p < 0.001). Particularly HEAS affective and behavioral symptoms were associated with lower levels of well-being.
Weiß et al. [70]	CCARPD	Depression and anxiety symptoms	CCAS scores were statistically significantly associated with GAD-7 (r = 0.56) and PHQ-2 (r = 0.42) scores.
Ali et al. [71]	CCARPD	Depression, anxiety, and stress symptoms	Correlations between HEAS subscales (“feelings of anxiety,” “rumination,” “behavioral symptoms,” and “anxiety about one’s impact”) and the DASS-8 depression, anxiety, and stress subscales ranged from low to moderate (from r = 0.17 to r = 0.43) (all p < 0.001).
Brailovskaia and Teismann [72]	CCARPD	Depression, suicidal ideation and entrapment	CCARPD is moderately associated with markers of negative mental health. An association between CCARPD and suicidal ideation was established, even after controlling for concurrent depressive symptoms. Entrapment was shown to completely mediate the association between CCARPD and suicidal ideation, pointing to the possibility that CCARPD is relevant to suicidal ideation only when it translates into perceptions of entrapment (the impression of being trapped in a hopeless situation). Thus, the present results point to entrapment as a significant psychological pathway connecting stressors and suicidal outcomes. CCARPD was statistically significantly (all p < 0.001) correlated with depression (r = 0.31), suicidal ideation (r = 0.28) and entrapment (r = 0.35).
Pitron et al. [73]	CCARPD	Depression, anxiety, somatic symptoms, and worries about health-related risks associated with environmental agents	CCARPD and symptoms of anxiety and depression were significantly positively correlated (r = 0.44, p < 0.001). 17% of the participants in the sample fulfilled the cut-off score PHQ-4 > 6, suggesting the existence of an anxiety or depressive disorder.
Tucholska et al. [74]	Neuroticism	CCARPD	CCAS indices of cognitive-emotional impairment (r = 0.16, p < 0.01) and functional impairment (r = 0.11, p < 0.05) are positively weakly related to neuroticism.
Hogg et al. [75]	CCARPD	Feelings of worry	CCARPD was related to feelings of worry. Correlations between HEAS subscales (“affective symptoms”, “rumination”, “behavioral symptoms”, and “anxiety about one’s impact”) and the PSWQ scale ranged from low to moderate (from r = 0.21 to r = 0.45) (all p < 0.001).
Fekih-Romdhane et al. [76]	CCARPD	Depression, anxiety, and stress symptoms	Higher CCARPD was statistically significantly associated with higher depression (r = 0.35), anxiety (r = 0.37) and stress (r = 0.24) (no p-values reported)
Carlson et al. [77]	CCARPD	Depression, anxiety, and stress symptoms	CCAS scores were moderately correlated with general levels of anxiety (n = 39, r = 0.49, p = 0.002) and stress (n = 39, r = 0.46, p = 0.003), and weakly correlated with depression (n = 39, r = 0.32, p = 0.04).
Zeier and Wessa [78]	CCARPD	Depression and anxiety symptoms	Moderate correlations (ranging from r = 0.40 to r = 0.44, p < 0.01) between EAQ-22 (negative consequences) or CCAS and psychological distress (PHQ-4 or PHQ-4 subscales general anxiety and depression) measurements.
Fekih-Romdhane et al. [79]	CCARPD	Death anxiety and prodromal psychotic symptoms	CCARPD has significant (p < 0.001) direct and indirect (via death anxiety as mediator) effects on psychotic symptoms.
Çimşir et al. [80]	CCARPD	Psychopathological symptoms (e.g., depression and anxiety symptoms)	“Behavioral symptoms”, “feelings of anxiety”, as well as “anxiety about one’s impact” dimensions of the HEAS-13 showed small-to-moderate correlations (ranging from 0.27 to 0.47, all p < 0.001) with clinically relevant psychological symptoms, while the “ruminative thoughts” dimension showed significant but smaller correlations (ranging from 0.13 to 0.22, p < 0.01 or p < 0.001) with the BSI symptoms.
Veijonaho et al. [81]	CCARPD	Depression symptoms	The overburdened group (the smallest group of participants [timepoint 1: 9% and timepoint 2: 10%]), representing an example of overwhelming CCARS and showing relatively high scores on all climate distress items, especially in the behavioral impairment items, experienced depression symptoms more at both time points, compared to the emotionally involved group (scoring lower in behavioral impairment-related items).
Orrù and Mannarini [82]	Emotion dysregulation	CCARPD	A statistical significant relationship (p < 0.01) between CCA and emotion dysregulation (r = 0.365) was found.
Lykins et al. [83]	CCARPD	Antenatal depression	Moderate positive association between CCAS and antenatal depression (r = 0.37, p < 0.001). CCARPD predicted 13.4% of the variance in antenatal depression scores (r = 0.38, r^2^ = 0.14, adj r^2^ = 0.13).
Hogg et al. [84]	CCARPD	Depression and anxiety symptoms	Correlations between HEAS subscales (“feelings of anxiety”, “rumination”, “behavioral symptoms”, and “anxiety about one’s impact”) and the GAD-7 or DASS-21 depression subscale ranged from moderate to strong (from r = 0.36 to r = 0.67) (all p < 0.001). Affective eco-anxiety was the strongest correlate of generalized anxiety and was associated with more symptoms of depression.
Wullenkord et al. [85]	Cumulative stressors	CCARPD	Exposure to cumulative stressors (i.e., low perceived presence of high-quality nature in the neighborhood, living in a large city, low education, unemployment, and low income) did not appear as a possible vulnerability factor for experiencing climate change anxiety-related impairment (r = 0.04).
Parmentier et al. [86]	Trait anxiety	CCARPD	Trait anxiety exhibited a positive and significant correlation with the climate anxiety-related functional impairment (r = 0.26) and cognitive-emotional impairment (r = 0.24) subscales of the CCAS (both p < 0.001). Trait anxiety has a minor role as a predictor of climate anxiety-related functional impairments, compared to other predictors.
Jalin et al. [87]	Environmental affect traits, media exposure, connectedness to nature, depression and anxiety symptoms	CCARPD	An increase in the EMEA global score was significantly associated with an increase in the anxiety score on the GAD-7 (b = 0.10; p < 0.05) and the depression score on the PHQ-9 (b = 0.09; p < 0.05). These links, however, were weak. 56% of the people in the eco-anxious group had GAD-7 scores corresponding to absent to mild anxiety.
Micoulaud-Franchi et al. [88]	CCARPD	Depression and anxiety symptoms	Correlation between EAQ-22 and HAD scores was low (r = 0.21, p < 0.001).
Er et al. [89]	CCARPD	Depression, anxiety, and stress symptoms	There is a moderate-to-high statistically significant relationship between the total and subscale scores of the HEAS and the total and subscale scores of the DASS-21 (correlations ranging from r = 0.39 to r = 0.58, all p < 0.01).
Nezlek and Cypryańska [90]	CCARPD	Depression and anxiety symptoms	Low-to-moderate correlations between CDS scores and scores on the GAD (r = 0.30) and CES-D (r = 0.23).
Plohl et al. [91]	CCARPD	Anxiety, neuroticism, well-being, and stress symptoms	Only weak negative associations between CCAS subscales and the emotional well-being subscale of the MCH-SF (r = −0.12 and r = −0.15, all p < 0.01), and low-to-moderate associations between CCAS subscales and anxiety and stress subscales of the DASS-21 or neuroticism subscale of the BSI-2-S (ranging from r = 0.21 to r = 0.30, all p < 0.001).
Asgarizadeh et al. [53]	Anxiety symptoms	CCARPD	Symptoms of general anxiety disorder were the most important direct predictor of CCARPD. GAD-7 scores were strongly associated with CCARPD (r = 0.60, p < 0.01).
Patrick et al. [92]	CCARPD	Pre-traumatic stress symptoms	Among participants who had not experienced a climate change event, nearly 16% met the screening criteria for pre-traumatic stress symptoms in relation to fears about future trauma related to climate change.
Thomson and Roach [93]	CCARPD	Depression, anxiety, and stress symptoms	The three psychological distress variables (depression, anxiety, and stress) were all moderately correlated with the CCAS total score (r = 0.46, r = 0.39, and r = 0.39, respectively, all p < 0.001).
Türkarslan et al. [6]	CCARPD	Depression, anxiety, and stress symptoms	Correlations between HEAS subscales (“feelings of anxiety”, “rumination”, “behavioral symptoms”, and “anxiety about one’s impact”) and the DASS-21 subscales (anxiety, depression, and stress subscales) ranged from weak-to-moderate (from r = 0.24 to r = 0.37, all p < 0.001).
Ediz and Yanik [94]	Climate change awareness	CCARPD and hopelessness	A significant relationship was identified between previous psychological support and the average score on the CCAS among climate activists (p = 0.008). Level of hopelessness was higher among climate activists who were more aware about climate change, compared to those who had partial knowledge (p = 0.002).
Wu et al. [51]	CCARPD	Depression and anxiety symptoms	Small but statistically significant positive correlations were found between CCAS-S and general anxiety (r = 0.17, p < 0.0001) and depression (r = 0.14, p < 0.0001).
Hepp et al. [52]	CCARPD	Depression and anxiety symptoms, neuroticism, and positive (e.g., enthusiastic) and negative (e.g., guilty) affect	Small positive correlations (varying between r = 0.02 and r = 0.20) between CCAS subscales (cognitive-emotional and functional impairment) and CCDIS impairment subscale and depressive symptoms (BDI-II), anxiety symptoms (GAD-7), neuroticism (NEO-FFI) and negative affect (PANAS-X).
Mathers-Jones and Todd [95]	CCARPD	Depression, anxiety, and stress symptoms	CCARPD was significantly positively correlated with depression (r = 0.36, p < 0.001), anxiety (r = 0.38, p < 0.001), and stress (r =0.27, p < 0.01).
Vercammen et al. [96]	Depression, anxiety, and stress symptoms	CCARPD	There was a significant overall effect of mental health status (p = 0.005) on climate distress. Those currently diagnosed and/or receiving treatment for a mental health condition were significantly more likely to experience moderate, as opposed to low climate distress (OR = 1.930), and high, as opposed to low climate distress (OR = 3.116).
Heinzel et al. [97]	CCARPD	Depression, anxiety, and stress symptoms	Correlations between HEAS subscales (“feelings of anxiety”, “rumination”, “behavioral symptoms”, and “anxiety about one’s impact”) and the DASS-21 subscales (anxiety, depression, and stress subscales) ranged from weak-to-moderate (from r = 0.23 to r = 0.49) (all p < 0.001).
Lutz et al. [55]	CCARPD	Depression, anxiety, and stress symptoms, and positive (e.g., enthusiastic) and negative (e.g., guilty) affect	CCA (mean 1.61 on a 5-point scale) was statistically significantly (all p < 0.001) correlated with depression (r = 0.27), anxiety (r = 0.34) and stress (r = 0.28). CCA was statistically significantly (p < 0.01) correlated with negative affect (r = 0.17).
Lukacs et al. [98]	CCARPD	Psychological distress	CCAS scores were strongly correlated with K6 scores (r = 0.70, p < 0.0001).
Mathé et al. [99]	CCARPD	Depression, anxiety, and stress symptoms	Small-to-moderate correlations were found between HEAS subscales (“feelings of anxiety”, “rumination”, “behavioral symptoms”, and “anxiety about one’s impact”) scores, DASS-21 subscale scores (anxiety, depression, and stress subscales, from r = 0.26 to r = 0.46, all p < 0.001), and the STAI-Y scores (from r = 0.29 to r = 0.48, all p < 0.001).
Innocenti et al. [100]	CCARPD	Depression, anxiety, and stress symptoms	There was no correlation between the HEAS or the CCAS and the subscales of the DASS–21 (stress, anxiety, and depression subscales)
Schwartz et al. [18]	CCARPD	Depression and anxiety symptoms	*Quantitative results* 28.9% and 37% were classified as having probable MDD and/or probable GAD (n = 47), respectively. Moderate correlations between both CCAS cognitive-emotional impairment and CCAS functional impairment subscales and MDD and GAD symptom severity (both, respectively, r = 0.24 and r = 0.28, p < 0.001). Higher levels on both CCAS cognitive-emotional impairment and CCAS functional impairment subscales were significantly associated with higher GAD symptoms (p = 0.046 and p = 0.011, respectively). Higher CCA functional impairment was significantly associated with higher MDD symptoms (p = 0.026), whereas the association between CCA cognitive-emotional impairment and MDD symptoms was nonsignificant (p = 0.131).
Heeren et al. [101]	CCARPD	General worry	Small correlations have been found between both CCAS cognitive-emotional impairment and CCAS functional impairment subscales and general worry.
Schwaab et al. [102]	CCARPD	Depression and anxiety symptoms, traumatic and perceived stress	Participants showed high prevalence of symptoms of depression, anxiety, and perceived stress. However, few participants showed moderate or severe symptoms of depressive disorder or general anxiety disorder, nor traumatic symptom burden in relation to climate change. The symptomatic burden with respect to climate change was restricted to considerable levels of perceived stress. Results mental health disorder measurements 42% showed mild to severe depressive symptoms 50% showed low to high levels of anxiety 10% had probable clinically significant traumatic symptom burden 27% had moderate or high levels of perceived stress 9% diagnosed with mental disorder 6% currently in psychotherapy Results modified climate change-related version of mental health disorder measurements 3% showed at least mild symptoms of depression associated with climate change 10% showed at least low levels of anxiety associated with climate change 1% was positively screened for climate change-related traumatic symptom burden 23% showed moderate or high levels of perceived stress as a consequence of climate change
Whitmarsh et al. [54]	Anxiety symptoms	CCARPD	Higher CCARPD (mean 1.25 on a 5-point scale = climate anxiety was low) was correlated with higher generalized anxiety (r = 0.23, p < 0.001). Increased generalized anxiety (β = 0.01, p < 0.001) uniquely predicted increased climate anxiety.
Feather and Williams [103]	Climate change concern	CCARPD and depression and anxiety symptoms	A small (r = 0.17) but significant (p < 0.001) correlation was found between CCARPD and anxiety and depression.
Hajek and König [104, 105]	CCARPD	Loneliness and perceived social isolation	Correlations between CCAS and anxiety and depressive symptoms were moderate (respectively, r = 0.31 and r = 0.30, all p < 0.001, with Bonferroni-adjusted significance level). Regressions showed that a higher CCA was associated with a higher likelihood of probable depression (OR: 1.37, 95% CI: 1.25–1.50) and of probable anxiety (OR: 1.27, 95% CI: 1.15–1.40) (both p < 0.001). CCA and loneliness were positively associated (small effect size, r = 0.19, p < 0.001). CCA and perceived social isolation were also positively associated (nearly medium effect size, r = 0.28, p < 0.001).
Mouguiama-Daouda et al. [106]	CCARPD	Depression and anxiety symptoms	Positive correlation between CCA and depression (r = 0.30, p < 0.05, corrected for multiple comparisons). No significant correlation between CCAS and generalized anxiety was found.
Larionow et al. [107]	CCARPD	Depression and anxiety symptoms	Overall CCAS score was statistically significantly related to depressive symptoms (r = 0.26, p < 0.01), but was not associated with anxiety symptoms (r = 0.12).
Cruz and High [108]	CCARPD	Depression and state and trait anxiety	Small-to-moderate correlations between CCA and depression (r = 0.30) and trait anxiety (r = 0.26).
Lawrance et al. [43]	CCARPD	Depression, anxiety, and stress symptoms	Individuals with high levels of generalized anxiety reported higher levels of distress for the climate crisis than respondents with no or low levels of anxiety.
Wullenkord et al. [109]	CCARPD	Depression and anxiety symptoms and basic psychological needs	The overall mean score on the CCA (mean 1.81 on a 7-point scale) correlated with “generalized anxiety and depressiveness” (r = 0.25, p < 0.01).
Hogg et al. [110]	CCARPD	Depression, anxiety, and satisfaction with life	Australian study Correlation between dimension “affective symptoms” and generalized anxiety was r = 0.38 (p < 0.001). New Zealand study *Sample 1* The eco-anxiety dimensions “feelings of anxiety” and “behavioral symptoms” were generally more strongly related to stress (r = 0.42 and r = 0.30, respectively), depression (r = 0.37 and r = 0.35, respectively) and anxiety (r = 0.46 and r = 0.31, respectively) (all p < 0.001) than the dimensions “rumination” (r = 0.22, r = 0.15 and r = 0.22, respectively) and “anxiety about one’s impact” (r = 0.27, r = 0.21 and r = 0.28, respectively) (all p < 0.001, except for correlation between “rumination” and depression [p < 0.01]). Eco-anxiety dimension “affective symptoms” has been found to be the only significant predictor of overall anxiety as measured by the DASS-anxiety subscale (β = 0.42, p < 0.001). *Sample 2* The eco-anxiety dimensions “feelings of anxiety” and “behavioral symptoms” were generally more strongly related to stress (r = 0.54 and r = 0.50, respectively), depression (r = 0.46 and r = 0.45, respectively) and anxiety (r = 0.59 and r = 0.45, respectively) (all p < 0.001) than the dimensions “rumination” (r = 0.33, r = 0.25 and r = 0.36, respectively) and “anxiety about one’s impact” (r = 0.50, r = 0.45 and r = 0.45, respectively) (all p < 0.001). Eco-anxiety dimension “affective symptoms” has been found to be the only significant predictor of overall anxiety as measured by the DASS-anxiety subscale (β = 0.52, p < 0.001).
Innocenti et al. [111]	CCARPD	Depression and anxiety symptoms	CCAS cognitive impairment subscale showed a positive correlation with GAD total score, K-10 total score, K-10 depression subscale, and K-10 anxiety subscale. Small positive association between the CCAS functional impairment subscale showed and the GAD total score, but not K10 anxiety subscale, K10 depression subscale, or K10 psychological distress subscale.
Clayton and Karazsia [112]	CCARPD	Depression and anxiety symptoms	CCAS [both cognitive (r = 0.54) and functional (r = 0.47) impairment subscales] was strongly associated with general anxiety/depression.

Abbreviations: BDI-II, Beck Depression Inventory-II; BFI-10, Short 10-item Big Five Inventory; BFI-15, Short 15-item Big Five Inventory; BHS, Beck Hopelessness Scale; BSI, Brief Symptom Inventory; CCA, Climate Change Anxiety; CCARS, Climate Change Awareness-Related Distress; CCAS, Climate Change Anxiety Scale; CCAS-S, Climate Change Anxiety Scale Short-Form (abbreviated 4-item version of the CCAS); CCDIS, Climate Change Distress and Impairment Scale; CDS, Climate Distress Scale; CES-D-10, Center for Epidemiologic Studies Depression Scale, 10-item version; CES-D-8, Center for Epidemiologic Studies Depression Scale, 8-item version; CSD, Cross-Sectional Design; DASS-8, Depression Anxiety Stress Scale, 8-item version; DASS-21, Depression Anxiety Stress Scale, 21-item version; DASS-42, Depression Anxiety Stress Scale, 42-item version; DCE, Discrete Choice Experiment; DEPS-10, The Depression Scale, 10-item version; DERS-SF, Difficulties in Emotion Regulation Scale – Short Form; DT, Distress Thermometer; EAQ-22, Eco-Anxiety Questionnaire; EID, Environmental Identity Scale; EMEA, Eco-Anxiety Measurement Scale; EPDS, Edinburgh Postnatal Depression Scale; GAD, General Anxiety Disorder; GAD-2: General Anxiety Disorder scale, 2-item version; GAD-4: General Anxiety Disorder scale, 4-item version; GAD-7, General Anxiety Disorder scale, 7-item version; GAD-7-C, The climate change specific General Anxiety Disorder scale, 7-item version; GAD-9, General Anxiety Disorder scale, 9-item version; HAD, Hospital Anxiety and Depression Scale; HEAS, Hogg Eco-Anxiety Scale; K-6, Kessler Psychological Distress Scale, 6-item version; K-10, Kessler Psychological Distress Scale, 10-item version; MDD, Major Depressive Disorder; MHC-SF, Mental Health Continuum-Short Form; MHW-12, Modern Health Worries scale (12 questions); MMS, Mixed-Methods Study; MRI, Magnetic Resonance Imaging; NEO-FFI, NEO Five-Factor Inventory; NR, Not Reported; PANAS-X, Positive and Negative Affect Schedule- expanded form; PHQ-4, Patient Health Questionnaire for depression and anxiety, 4-item version; PHQ-8, Patient Health Questionnaire for depression, 8-item version; PHQ-9, Patient Health Questionnaire for depression, 9-item version; PHQ-9-C, The climate change specific Patient Health Questionnaire for depression, 9-item version; PMS, Premenstrual Syndrome; PQ-B, Prodromal Questionnaire-Brief; PSIS, Perceived Social Isolation Scale; PSS-4, Perceived Stress Scale, 4-item version; PSWQ, Penn State Worry Questionnaire; PSQ-20, Perceived Stress Questionnaire, 20-item version; PSQ-20-C, the climate change specific Perceived Stress Questionnaire, 20-item version; PTSD-8, Post-Traumatic Stress Disorder, 8-item questionnaire; PTSS-10, Posttraumatic Stress Scale, 10-item version; PTSS-10-C, The climate change specific Posttraumatic Stress Scale, 10-item version; QDIA-SR, Quick Depression Inventory for Adolescents-Self-Report Form; SDA, Scale of Death Anxiety; SDES, Short Defeat and Entrapment Scale; SDQ, Strengths and Difficulties Questionnaire; SIBS, Suicide Ideation and Behavior Scale; SPANE, Scale of Positive and Negative Experiences; SSS-8, Somatic Symptoms Scale (8 categories of somatic symptoms); STAIT-5, State-Trait Anxiety Inventory Scale-5 questionnaire; STAI-Y, Spielberger Trait Anxiety Scale-Form Y; UK, United Kingdom; US, United States; WHO-5, World Health Organization-Five Well-Being Index.

**Table 4.** Results of studies in the clinical population [27, 113, 114]

Author(s)	Country	Study design	N	Psychiatric diagnosis	Mean age (SD), range (years)	Female (%)	Scales	Results
Lerolle et al. [113]	France	CSD	87	Anorexia nervosa, n = 12; bulimia nervosa, n = 1; gender dysphoria, n = 1; major depressive episode, n = 18; emerging psychosis, n = 9; anxious school refusal, n = 1; PTSD, n = 3; isolated suicide attempt, n = 16; generalized anxiety disorder, n = 1; bipolar disorder, n = 4; borderline disorder, n = 4; behavior disorder, n = 3; ADHD, n = 7; OCD, n = 3; ASD, n = 4	13.8 (1.1), 12–16	82.8	CCAS C-SSRS HAD	Although the CCAS total score was statistically significantly associated with the C-SSRS total score (p < 0.05), the cognitive/emotional and functional impairment components of CCAS were not associated with the severity of suicide risk. Correlations between CCAS total score or the cognitive/emotional and functional impairment components of CCAS with the HAD were low (from r = 0.16 to r = 0.27).
Gebhardt et al. [114]	Germany	CSD	89	Depression, n = 56; anxiety disorder, n = 19; PTSD, n = 14; missing, n = 10) (multiple diagnoses were possible)	37.6 (15.1), NR	68	PHQ-9/PHQ-9-C GAD-7/GAD-7-C PTSS-10/PTSS-10-C	Moderate positive association between climate change awareness-related anxiety symptoms (GAD-7-C) and anxiety symptoms (GAD-7) (r = 0.41, p < 0.001). Overall, 11% of the participants reported mild depressive symptoms due to climate change awareness; 20% mild anxious symptoms (and 1% moderate levels of anxiety); no participant reported traumatic symptoms due to climate change awareness.
Jones et al. [27]	Australia	Naturalistic study	50	DSM-IV-TR diagnosis OCD using the CIDI-Auto v2.1	38 (14.7), 19–86	42	Y-BOCS PI-WSUR VOCI BDI DASS-21	28% of the participants were identified as having OCD climate change-related concerns (OCD CCC group) (the majority of these participants were male: 71%). There were no significant differences between the OCD CCC group and the OCD non-CCC group on any of the measures.

Abbreviations: ADHD, Attention Deficit Hyperactivity Disorder; ASD, Autism Spectrum Disorder; BDI, Beck Depression Inventory; CIDI-Auto, The Composite International Diagnostic Interview; C-SSRS, Columbia-Suicide Severity Rating Scale; DASS-21, Depression Anxiety Stress Scale 21; GAD-7, General Anxiety Disorder scale, 7-item version; GAD-7-C, The climate change specific General Anxiety Disorder scale, 7-item version; HAD, Hospital Anxiety and Depression Scale; HEAS, The Hogg Eco-Anxiety Scale; MDD, Major Depressive Disorder; N/A, Not Applicable; OCD, Obsessive Compulsive Disorder; PHQ-9, Patient Health Questionnaire for depression, 9-item version; PHQ-9-C, The climate change specific Patient Health Questionnaire for depression, 9-item version; PTSD, Post-Traumatic Stress Disorder; PTSS-10, Posttraumatic Stress Scale, 10-item version; PTSS-10-C, The climate change specific Posttraumatic Stress Scale, 10-item version; PI-WSUR, Padua Inventory – Washington State University Revision; VOCI, Vancouver Obsessional Compulsive Inventory; Y-BOCS, Yale-Brown Obsessive Compulsive Scale.

### Association between CCARPD and mental health in people with psychiatric diagnoses or subclinical symptoms

#### General population studies

The available evidence shows that in most general population studies [6, 18, 43, 51, 52, 54, 55, 58–61, 64, 65, 67–78, 80, 82, 83, 85–88, 90, 93, 95, 99, 103–111] significant positive correlations were found between CCARPD and mental health measurements. These correlations, however, were mostly low (r < 0.30) to moderate (r = 0.30–0.49) (see Table 3). Trait anxiety, a personality trait to perceive various events as threatening [115], also seems to play a minor role as a predictor of climate anxiety-related impairments [86].

Studies in general populations have also found that, although individuals at risk for mental illness may experience CCARPD, this distress rarely results in significant functional impairment. Only a minority of these individuals exhibited moderate or severe levels of distress in relation to climate change awareness [18, 51, 52, 54, 102]. Those who had a clinical history (i.e. previously receiving psychological support or having a mental health disorder) tended to experience higher distress levels due to the awareness of climate change [94, 96]. Vercammen et al. [96], for example, found in their online survey that the subgroup of those currently diagnosed and/or receiving treatment for a mental health condition were significantly more likely to experience moderate (OR = 1.930) and high levels (OR = 3.116) of CCARPD.

#### Psychiatric population studies

We found a paucity of literature examining the association between CCARPD and the mental health of people with a preexisting psychiatric diagnosis within clinical populations. Only 3 out of the 67 identified records concerned psychiatric populations (see Table 4).

These studies [27, 113, 114] illustrate how CCARPD can have an impact on the content of delusions held by people with a schizophrenia-spectrum disorder or a major depressive disorder with psychotic symptoms, or on the types of obsessions and compulsions experienced by people with obsessive-compulsive disorders. Jones et al. [27] found that 28% of their obsessive-compulsive patients had obsessions and compulsions directly related to climate change (such as the obsession that increased air temperatures would result in rapid evaporation of the water in their pet bowls, leading to their pets dying of thirst, followed by checking and rechecking the pet’s water bowl to ensure they held adequate water).

The type of cognitive biases contributing to depressive, anxiety or PTSD symptoms seems to be related to the type of climate change awareness-related symptoms. The study of Gebhardt et al. [114], for example, showed patients with depression might focus more on the sadness-related feelings evoked by climate change awareness, whereas those with anxiety disorder or PTSD rather had threat-related feelings evoked by climate change awareness.

## Discussion

The positive, but mostly small-to-moderate, correlations between CCARPD and mental health measurements (anxiety, depressive, or stress symptoms) that have been observed across general and psychiatric population studies are in line with those of other recent reviews with distinct research questions and methods [116, 117]. The systematic review of Cosh et al. [117] reported small-to-large positive correlations (r = 0.14 to r = 0.60) between eco-anxiety and depressive, anxiety, or stress symptoms in general population and mental health settings. Eco-anxiety in this review, however, was broadly defined and also included environmental worry, probably explaining why associations with mental health outcomes were more variable than in our review. Moreover, probably partly due to a less extensive and updated search strategy, only 35 studies were included in their review. Gago et al. [116], finding a moderate negative correlation (r = −0.30) between eco-anxiety and psychological well-being, limited their analyses to eco-anxiety as measured by the CCAS. Despite this, all these results suggest that people with mental health vulnerabilities may be more prone to experience higher levels of CCARPD and functional impairment. However, as these data are cross sectional, they are insufficient to establish a causal relationship between potential or preexisting psychopathology and higher levels of CCARPD. It is plausible that people with mental health vulnerabilities look at climate change-related events with a general negative bias, leading to higher states of anxiety, depression, and/or stress. On the other hand, it is also likely that the awareness of climate-related changes is potentially stressful and detrimental to overall mental health and particularly might be a risk factor for these individuals. The resulting negative thoughts and feelings may as such contribute to the exacerbation or even the development of psychiatric disorders. There might also be a bidirectional association between both variables [21, 22, 75, 78, 89, 118–120].

There is still little consensus on how to conceptualize CCARPD from a mental health standpoint [18, 121]. Most authors, mental health professionals, and climate scientists [6–9, 15, 17, 37, 43, 77, 81, 84, 90, 92, 97, 110, 116, 122–130] agree that emotional reactions to climate change should not be pathologized and can be seen as an adequate, adaptive, functional, justifiable, and rational response to the serious threats facing the planet and its inhabitants. Moreover, some [131] have even argued that it might be pathological to have too little CCARPD, given the urgency of climate action.

There is no doubt that climate change poses an existential threat to humans and other living organisms [132, 133]. The global ecological crisis has indeed been related to many existential concerns and deep feelings of ontological insecurity [109, 129, 134, 135]. This existential threat challenges prevailing values, ethics, and way of thinking and imposes a set of constraints on human behavior (such as [mental] health and forced migration) and basic preconditions (such as food security) [55, 125, 133]. Given its existential nature, it is not surprising and quite plausible that CCARPD is experienced by numerous people who do not suffer from mental health issues.

However, irrespective of whether it represents a rational concern, results highlight that CCARPD may also be overwhelming, “irrationally high,” excessive, and uncontrollable, and may reach levels that interfere with an individual’s ability to function, especially for people with underlying symptoms of anxiety, depression, or stress [6, 7, 9, 15, 17, 18, 37, 39, 63, 81, 85, 92, 97, 112, 116, 125, 128, 136, 137].

Several studies found a strong association between CCARPD and pro-environmental attitudes or behaviors, even when controlling for several correlates of environmental behavior (e.g., political ideology) [18, 74, 84, 109, 110, 130, 138], suggesting that CCARPD may be a constructive form of distress and stimulate individuals to adopt behaviors that can counteract climate change (identified as the concerned steward effect or a coping mechanism) [98, 100, 109]. Thus, it seems that low-to-moderate levels of CCARPD (particularly anxiety symptoms) may be essential and required to take ecological problems seriously and engage in pro-environmental behaviors. High or excessive levels of CCARPD, however, can have a paralyzing effect on pro-environmental behaviors [100, 101, 109, 137]. Heeren et al. [101] have shown that the relationship between CCARPD and pro-environmental behavior is weakest in individuals with the highest levels of CCARPD, suggesting that CCARPD in cases of high anxiety levels is not adaptive. Symptoms of CCARPD may also be more pronounced in individuals who lack emotional regulation skills. Emotional regulation is the individual’s ability to identify and regulate the emotions they have. In those with high levels of emotion dysregulation, climate change awareness-related anxiety may reach levels that deteriorate daily functioning, predicting worse mental health outcomes [82].

An important question here is whether CCARPD also deserves a special status (as a separate disorder) in the existing psychiatric classifications [139]. Some health professionals, for example, see CCARPD as a form of “pre-traumatic stress disorder,” in which traumatic consequences are anticipated and felt (i.e., pre-traumatic stress symptoms, including patterns of avoidance and symptoms of hypervigilance) before the event even takes place [29, 92, 140]. However, until now, current diagnostic manuals (i.e., the DSM-5-TR and ICD-11) do not include ecological disorders such as eco-anxiety or climate change awareness-related phobia, depression, or obsessions/compulsions [136].

Given that various environmental issues are expected to worsen and humanity does not presently seem able to stop it effectively, CCARPD is likely to become a greater focus for mental health professionals and psychotherapists in the coming years [17, 29, 37, 55, 141]. Mental health professionals and psychotherapists already are increasingly reporting counseling individuals dealing with CCARPD [9, 37, 142, 143]. In a nationwide sample of German psychotherapists (N = 573), for example, about 72% of them indicated having patients with climate change-related concerns [37]. Findings suggest, however, that these issues are not yet adequately addressed in psychotherapy [144]. Psychotherapists therefore should be prepared to address them.

The development of psychotherapeutic interventions to address the adverse mental health effects of climate changes have already been indicated by the American Psychiatric Association and the European Psychiatric Association as a legitimate focus of psychiatrists and psychologists [145–147], hereby emphasizing a salutogenic (i.e., identifying and promoting factors that contribute to well-being), rather than a pathogenic approach [92, 123]. Acceptance and commitment therapy, for example, may be appropriate, as it focuses on validating experienced emotions and changing the individual’s relationship to their thoughts and cognitions, rather than questioning their validity. It also encourages patients to be fully engaged and present in the current moment, rather than dwelling on the past (e.g., eco-grief) or worrying about the future (e.g., eco-anxiety), and to take action to address environmental issues [17, 84, 136]. According to Hogg et al. [84], components of Dialectical Behavior Therapy that help people develop insight into their CCARPD (e.g., what triggers it, how it manifests, what its function is) may also indirectly benefit well-being. Several other therapies, including environmental identity-based therapies [148], nature-based art therapy [149], and the CliMACT training [150], are explored to see how they can support people with CCARPD within the context of the ecological crisis. Interventions involving cognitive reappraisal of anxiety-provoking objects as less threatening, on the other hand, may not be appropriate given the genuine threat of climate change [95]. Making individual behavioral changes to cut one’s global footprint may be seen as another way of decreasing climate change anxiety because the person now feels that they are helping to address the problem. However, for people with obsessive compulsive disorder, it is possible that this approach can actually exacerbate the anxiety and contribute to further checking [27].

This scoping review identified several research gaps. First, concept and terminology of CCARPD needs to be better defined [48, 82, 151], as it may restrict replicability and comparability of studies [151]. There exists still no formal definition of CCARPD [142]. According to Orrù and Mannarini [82] more than ten distinct operationalizations of this concept are currently used in the literature. This is reflected in the diverse ranges of measures, complicating efforts to compare findings across studies [48, 151]. Second, more research on the association between CCARPD and mental health in people is necessary, particularly in clinical populations and young people. Moreover, future research should also examine how one can help these people cope with negative emotions about climate change and explore the role of mental practitioners herein. Third, most of the conducted studies have a cross-sectional design. This design cannot be used to infer causality between CCARPD and mental health. It is therefore impossible to understand how CCARPD and mental health affect each other over time [152]. Longitudinal studies to assess the direction(s) of causality between CCARPD and mental disorders therefore are needed, as well as specific studies examining the role of coping strategies (coping strategies people use to reduce their anxiety over time cannot be uncovered in cross-sectional designs) [109]. Fourth, several studies, particularly those in the United States and Australia, may have some bias selection toward direct and/or indirect consequences of climate change. As particularly these areas are affected by climate change such as bushfires, floods, and extreme heat waves, one can wonder whether the included studies only measured climate change awareness or also direct and indirect effects of climate change. A nation-wide Australian study [92], approximately representative of adults in Australia, showed that in their large sample the majority of Australians (54.63%) already had a direct experience of a climate change-related event (e.g., directly affected by bushfire, flood, extreme heat wave). Another limitation is the methodological problem of self-report bias in the selected studies. It is important to know that all psychological scales that have been used in non-clinical population studies are self-report measures of anxiety or depressive symptoms and only indicative for clinically confirmed psychiatric diagnoses such as generalized anxiety disorder. Respondents may also underreport symptoms of mental health disorders, potentially weakening study measures [153]. Future general population studies therefore should try to use other measures, such as structural mental health interviews [51]. Finally, studies examining the association between CCARPD and mental health also fail to explicitly account for differences in the content and valence of climate change information conveyed to audiences by the media. Not only the mere volume of media exposure, but also the content of the information and the amount of attention people pay to it is important. Some studies suggest that media attention may be a better predictor of CCARPD than media exposure alone [54, 154].

A key strength of this review certainly is the extensive search strategies, including several databases (see Supplementary Material) to identify relevant studies on this topic. The search was also performed without language restrictions. Although we included studies with a mixed methods design, a major limitation is that our primary goal was to collect and analyze numerical, quantitative data. The inclusion of more qualitative data probably would have provided more in-depth, rich insights into the complex associations between CCARPD and mental health.

## Conclusion

Although CCARPD in most cases represents a reasonable and comprehensible reaction to serious environmental issues, our review shows that it can still be dysfunctional for people with mental health vulnerabilities, sometimes requiring professional support. As CCARPD will increase globally as the climate crisis unfolds in the coming decades while the understanding of the connections between CCARPD and mental well-being is still at an early stage of development, more research will be of utmost relevance. Particularly research in psychiatric populations is urgently needed to investigate how CCARPD may contribute to the exacerbation or reemergence of preexisting psychiatric disorders and how standard psychotherapeutic interventions should be adapted to address CCARPD in these populations successfully. In this regard, the development of clinical cut-off scores for CCARPD (such as these proposed by the systematic review of Cosh et al. [63], i.e., ≥21 on the CCAS for mild-moderate CCARPD, and ≥23 on the CCAS for severe-extremely severe CCARPD) may be opportune to guide clinical practice.

## Supporting information

10.1192/j.eurpsy.2026.10169.sm001 Peuskens et al. supplementary material Peuskens et al. supplementary material

## Data Availability

The search strings are available as supplementary material.

## Footnotes

1
https://blogs.lshtm.ac.uk/library/2018/12/07/removing-duplicates-from-an-endnote-library/. Last Access August 5, 2025.

## Supplementary material

The supplementary material for this article can be found at http://doi.org/10.1192/j.eurpsy.2026.10169.
