# Supplementary material for: Association between climate change awareness-related psychological distress and mental health in people with psychiatric diagnoses or subclinical symptoms: a scoping review
Source: Eur Psychiatry. 2026 Feb 18;69(1):e35. doi: 10.1192/j.eurpsy.2026.10169 (PMC13122536; doi:10.1192/j.eurpsy.2026.10169)
Supplement: Peuskens et al. supplementary material [file S0924933826101692sup001.docx]

**PUBMED**

("Anxiety Disorders"[Mesh:NoExp] OR "Neurotic Disorders"[Mesh] OR "Obsessive-Compulsive Disorder"[Mesh:NoExp] OR “Obsessive Behavior”[Mesh:NoExp] OR "Panic Disorder"[Mesh] OR "Phobic Disorders"[Mesh:NoExp] OR "Psychological Distress"[Mesh] OR "Catastrophization"[Mesh:NoExp] OR "anxiet*"[tiab] OR "neurotic*"[tiab] OR "neurosis"[tiab] OR "neuroses"[tiab] OR "psychoneurosis"[tiab] OR "psychoneuroses"[tiab] OR “psychoneurotic*”[tiab] OR "obsessive*"[tiab] OR "compulsive*"[tiab] OR "obsession*"[tiab] OR "compulsion*"[tiab] OR "OCD"[tiab] OR "panic"[tiab] OR “panics”[tiab] OR “panicks”[tiab] OR “panicking”[tiab] OR “panicked”[tiab] OR "phobi*"[tiab] OR "catastrophizing*"[tiab] OR "catastrophising*"[tiab] OR "catastrophisation*"[tiab] OR "catastrophization*"[tiab] OR "catastrophic thinking*"[tiab] OR "Rumination, Cognitive"[Mesh] OR "rumination*"[tiab] OR "ruminative thinking*"[tiab] OR "psychological distress*"[tiab] OR "emotional distress*"[tiab] OR "emotional stress*"[tiab] OR "distress syndrome*"[tiab] OR "repetitive behavior*"[tiab] OR "repetitive behaviour*"[tiab] OR "Mood Disorders"[Mesh] OR "Depression"[Mesh] OR "Premenstrual Syndrome"[Mesh] OR "depression"[tiab] OR "depressions"[tiab] OR “depressive”[tiab] OR “depressed”[tiab] OR "unipolar disorder*"[tiab] OR "MDD"[tiab] OR "disruptive mood dysregulation disorder*"[tiab] OR "dysthymi*"[tiab] OR "disthymi*"[tiab] OR "dysthimi*"[tiab] OR “premenstrual syndrome*”[tiab] OR "premenstrual dysphoric disorder*"[tiab] OR "Schizophrenia Spectrum and Other Psychotic Disorders"[Mesh] OR "schizophreni*"[tiab] OR "schizoaffective*"[tiab] OR "schizo-affective*"[tiab] OR "delusional"[tiab] OR "paranoid"[tiab] OR "schizotypal"[tiab] OR "psychotic*"[tiab] OR "psychosis"[tiab] OR "psychoses"[tiab] OR "catatoni*"[tiab] OR "bipolar*"[tiab] OR "cyclothymi*"[tiab] OR "Mania"[Mesh] OR "manic"[tiab] OR "hypomanic"[tiab] OR "hypomania*"[tiab] OR "mania"[tiab] OR "affective disorder*"[tiab] OR "mood disorder*"[tiab] OR "Autism Spectrum Disorder"[Mesh] OR "Attention Deficit Disorder with Hyperactivity"[Mesh] OR "autism*"[tiab] OR "autistic*"[tiab] OR "Asperger*"[tiab] OR "pervasive developmental disorder*"[tiab] OR "pervasive child developmental disorder"[tiab:~0] OR "pervasive child developmental disorders"[tiab:~0] OR "PDD"[tiab] OR "ASD"[tiab] OR "attention deficit disorder*"[tiab] OR "attention deficit hyperactivity disorder*"[tiab] OR "attention deficit and disruptive behaviour disorder*"[tiab] OR "attention deficit and disruptive behavior disorder*"[tiab] OR "ADHD"[tiab] OR "PDDNOS"[tiab] OR "Stress Disorders, Traumatic"[Mesh] OR trauma*[tiab] OR psychotrauma*[tiab] OR “emotional damage”[tiab] OR “emotional harm”[tiab] OR “emotional injur*”[tiab] OR “mental damage”[tiab] OR “mental harm”[tiab] OR “mental injur*”[tiab] OR “psychic damage”[tiab] OR “psychic harm”[tiab] OR “psychic injur*”[tiab] OR “psychological damage”[tiab] OR “psychological harm”[tiab] OR “psychological injur*”[tiab] OR “battered child syndrome*”[tiab] OR "combat disorder*"[tiab] OR "combat stress disorder*"[tiab] OR "shell shock*"[tiab] OR "PTSD"[tiab] OR “posttraumatic stress*”[tiab] OR “posttraumatic syndrome*”[tiab] OR "stress disorder*"[tiab] OR "acute stress*"[tiab] OR "chronic stress*"[tiab] OR “reactive attachment disorder*”[tiab] OR “severe stress*”[tiab] OR "Personality Disorders"[Mesh] OR "personalit*"[tiab] OR "borderline"[tiab] OR "antisocial"[tiab] OR "anti social"[tiab] OR "histrionic"[tiab] OR "narcissis*"[tiab] OR “narcism”[tiab] OR “narcist*”[tiab] OR "avoidant disorder*"[tiab] OR "schizoid"[tiab] OR sociopath*[tiab] OR psychopath*[tiab] OR dyssocial[tiab] OR dissocial[tiab] OR “social behavior disorder*”[tiab] OR “social behaviour disorder*”[tiab] OR “character disorder*”[tiab] OR “anankastic”[tiab] OR “negativistic”[tiab] OR “sadistic”[tiab] OR “masochistic”[tiab] OR “self defeating”[tiab] OR "Substance-Related Disorders"[Mesh] OR "Behavior, Addictive"[Mesh:NoExp] OR “addict*”[tiab] OR "Technology Addiction"[Mesh] OR "Gambling"[Mesh] OR "substance related disorder*"[tiab] OR "substance abuse*"[tiab] OR "drug abuse*"[tiab] OR "drug misuse*"[tiab] OR "substance use*"[tiab] OR "drug use*"[tiab] OR "drug habituation"[tiab] OR "drug bombing"[tiab:~0] OR "drug parachuting"[tiab:~0] OR "substance induced disorder*"[tiab] OR "substance dependen*"[tiab] OR "drug related disorder*"[tiab] OR "drug induced disorder*"[tiab] OR "drug dependen*"[tiab] OR "alcohol related disorder*"[tiab] OR "alcohol use disorder*"[tiab] OR "alcohol induced disorder*"[tiab] OR "alcoholism"[tiab] OR “alcoholic*”[tiab] OR “alcohol problem*”[tiab] OR “alcohol abuse*”[tiab] OR "alcohol dependen*"[tiab] OR "binge drinking"[tiab] OR "binge alcohol consumption"[tiab] OR “heavy drinking”[tiab] OR “excessive drinking”[tiab] OR “hazardous drinking”[tiab] OR “problem drinking”[tiab] OR “problematic drinking”[tiab] OR "caffeine"[tiab] OR "cannabis"[tiab] OR "marijuana"[tiab] OR "cocaine"[tiab] OR "amphetamine*"[tiab] OR "narcotic*"[tiab] OR "hallucinogen*"[tiab] OR "phencyclidine"[tiab] OR "inhalant*"[tiab] OR “glue abuse*”[tiab] OR “glue sniffing”[tiab:~0] OR “solvent abuse*”[tiab] OR “solvent sniffing”[tiab:~0] OR "solvent related disorder*"[tiab] OR "solvent dependen*"[tiab] OR "solvent use disorder"[tiab:~0] OR "solvent use disorders"[tiab:~0] OR "solvent induced disorder"[tiab:~0] OR "solvent induced disorders"[tiab:~0] OR "opioid*"[tiab] OR "opiate*"[tiab] OR "heroin"[tiab] OR "morphine"[tiab] OR "opium"[tiab] OR “anxiolytic*”[tiab] OR "hypnotic*"[tiab] OR "benzodiazepine*"[tiab] OR "sedative*"[tiab] OR tranquilizer*[tiab] OR tranquiliser*[tiab] OR "stimulant*"[tiab] OR “methamphetamine*”[tiab] OR "tobacco related disorder*"[tiab] OR "tobacco use disorder*"[tiab] OR "tobacco induced disorder"[tiab:~0] OR "tobacco induced disorders"[tiab:~0] OR "tobacco dependen*"[tiab] OR “tobacco abuse*”[tiab] OR "nicotine"[tiab] OR "gaming disorder*"[tiab] OR "game dependen*"[tiab] OR "videogame dependence"[tiab:~0] OR "videogame dependent"[tiab:~0] OR "gaming dependen*"[tiab] OR "compulsive gaming"[tiab] OR "compulsive computer gaming"[tiab:~0] OR "compulsive internet gaming"[tiab:~0] OR "compulsive video gaming"[tiab:~0] OR "compulsive videogaming"[tiab:~0] OR "pathological gaming"[tiab] OR "pathological computer gaming"[tiab] OR "pathological internet gaming"[tiab] OR "pathological video gaming"[tiab] OR "pathological videogaming"[tiab:~0] OR "excessive gaming"[tiab] OR "excessive computer gaming"[tiab] OR "excessive internet gaming"[tiab] OR "excessive video gaming"[tiab] OR "excessive videogaming"[tiab:~0] OR "internet use disorder*"[tiab] OR "internet-related disorder*"[tiab] OR “problematic computer use”[tiab] OR “pathological computer use”[tiab:~0] OR "problematic internet use"[tiab] OR “pathological internet use”[tiab] OR "internet dependen*"[tiab] OR "gambling disorder*"[tiab] OR "pathological gambling"[tiab] OR “problem gambling”[tiab] OR “problematic gambling”[tiab] OR “social media disorder*”[tiab] OR “problematic social media use”[tiab] OR “pathological social media use”[tiab:~0] OR “social media use disorder*”[tiab] OR "problematic use of social media"[tiab] OR "problematic screen use"[tiab] OR “problematic mobile phone use”[tiab] OR “problematic smart phone use”[tiab] OR “problematic mobilephone use”[tiab:~0] OR “problematic smartphone use”[tiab] OR “problematic use of mobile phone*”[tiab] OR “problematic use of smart phone”[tiab:~0] OR “problematic use of smart phones”[tiab:~0] OR “problematic use of mobilephone”[tiab:~0] OR “problematic use of mobilephones”[tiab:~0] OR “problematic use of smartphone*”[tiab] OR "severe mental illness*"[tiab] OR "serious mental illness*"[tiab] OR "severe mental disorder*"[tiab] OR "serious mental disorder*"[tiab]) AND ("Climate Change"[Mesh] OR "Greenhouse Effect"[Mesh] OR “greenhouse effect”[tiab] OR "climate change*"[tiab] OR "changing climate"[tiab] OR "climate emergenc*"[tiab] OR "climate impact*"[tiab] OR "climate-related impact*"[tiab] OR "global warming"[tiab] OR "ecological crisis"[tiab] OR "ecological threat*"[tiab] OR "climate crisis"[tiab] OR "climate disaster*"[tiab] OR "environmental disaster*"[tiab] OR "ecological disaster*"[tiab] OR "ecological loss"[tiab] OR "ecological problem*"[tiab] OR "environmental problem*"[tiab] OR "environmental crisis"[tiab] OR "environmental change*"[tiab] OR "environmental threat*"[tiab] OR “climate threat*”[tiab] OR "environmental destruction*"[tiab] OR “ecological destruction*”[tiab] OR "environmental issue*"[tiab] OR "ecological issue*"[tiab] OR “climate issue*”[tiab] OR "climate variability"[tiab] OR "environmental distress*"[tiab] OR "environmentally-induced distress"[tiab:~0] OR "eco-distress*"[tiab] OR “ecodistress*”[tiab] OR “ecological distress*”[tiab] OR “climate distress*”[tiab] OR “climate-related distress*”[tiab] OR "climate psychology"[tiab] OR "environmental psychology"[tiab] OR "eco-psychology"[tiab] OR "ecopsychology"[tiab] OR "eco-sickness"[tiab:~0] OR "ecosickness"[tiab] OR "eco-anxiety"[tiab] OR "ecoanxiety"[tiab] OR "eco-angst"[tiab:~0] OR "climate anxiet*"[tiab] OR "climate-related anxiety"[tiab:~0] OR “environmental anxiet*”[tiab] OR "ecophobia"[tiab] OR "eco-phobia"[tiab:~0] OR "eco-paralysis"[tiab] OR "ecoparalysis"[tiab] OR "eco-fear"[tiab:~0] OR “eco-fears”[tiab:~0] OR "ecofear*"[tiab] OR "eco-guilt"[tiab] OR "ecoguilt"[tiab] OR “environmental guilt”[tiab] OR "eco-shame"[tiab:~0] OR "ecosham*"[tiab] OR "ecological stress*"[tiab] OR "pre-traumatic stress*"[tiab] OR "anthropocene disorder"[tiab:~0] OR "anthropocene disorders"[tiab:~0] OR "psychoterratic*"[tiab] OR "ecological anxiety"[tiab:~0] OR "climate guilt"[tiab:~0] OR "climate-related guilt"[tiab:~0] OR "climate fear*"[tiab] OR "climate-related fear"[tiab:~0] OR "climate-related fears"[tiab:~0] OR "ecological worry"[tiab:~0] OR "ecological worries"[tiab:~0] OR “environmental worr*”[tiab] OR "ecotrauma*"[tiab] OR "solastalgia"[tiab] OR "eco-nostalgia"[tiab:~0] OR "econostalgia"[tiab] OR "environmental melancholy"[tiab:~0] OR "environmental melancholia"[tiab:~0] OR "eco-depression"[tiab] OR "ecodepression"[tiab] OR "eco-despair"[tiab:~0] OR "ecodespair"[tiab] OR "eco-anger"[tiab] OR "eco-overwhelm"[tiab:~0] OR "Environmentalism"[Mesh] OR “environmentalis*”[tiab] OR “environmental advocac*”[tiab] OR “climate advocac*”[tiab] OR “ecological advocacy”[tiab:~0] OR “ecological advocacies”[tiab:~0] OR “eco-advocacy”[tiab:~0] OR “eco-advocacies”[tiab:~0] OR "climate activis*"[tiab] OR "environmental activis*"[tiab] OR “ecological activist”[tiab:~0] OR “ecological activists”[tiab:~0] OR “ecological activism”[tiab:~0] OR “eco-activist”[tiab:~0] OR “eco-activists”[tiab:~0] OR “eco-activism”[tiab:~0] OR "pro-environmental behavior*"[tiab] OR "pro-environmental behaviour*"[tiab] OR "proenvironmental behavior*"[tiab] OR "proenvironmental behaviour*"[tiab] OR "ecological behavior*"[tiab] OR "ecological behaviour*"[tiab])

**EMBASE**

('anxiety disorder'/de OR 'neurosis'/exp OR 'acute stress disorder'/exp OR 'catastrophizing'/de OR 'distress syndrome'/exp OR 'generalized anxiety disorder'/exp OR 'obsessive compulsive disorder'/de OR 'compulsion'/exp OR 'obsession'/de OR 'refractory obsessive compulsive disorder'/exp OR 'panic'/exp OR 'phobia'/de OR 'posttraumatic stress disorder'/exp OR ‘posttraumatic stress*’:ti,ab,kw OR ‘posttraumatic syndrome*’:ti,ab,kw OR 'battered child syndrome'/exp OR ‘battered child syndrome*’:ti,ab,kw OR ‘combat disorder*’:ti,ab,kw OR ‘combat stress disorder*’:ti,ab,kw OR ‘shell shock*’:ti,ab,kw OR 'psychotrauma'/exp OR ‘trauma*’:ti,ab,kw OR ‘psychotrauma*’:ti,ab,kw OR 'emotional damage':ti,ab,kw OR ‘emotional harm’:ti,ab,kw OR 'emotional injur*':ti,ab,kw OR 'mental damage':ti,ab,kw OR 'mental harm':ti,ab,kw OR 'mental injur*':ti,ab,kw OR 'psychic damage':ti,ab,kw OR ‘psychic harm’:ti,ab,kw OR 'psychic injur*':ti,ab,kw OR 'psychological damage':ti,ab,kw OR 'psychological harm':ti,ab,kw OR 'psychological injur*':ti,ab,kw OR 'anxiet*':ti,ab,kw OR 'neurotic*':ti,ab,kw OR 'neurosis':ti,ab,kw OR 'neuroses':ti,ab,kw OR 'psychoneurosis':ti,ab,kw OR 'psychoneuroses':ti,ab,kw OR ‘psychoneurotic*’:ti,ab,kw OR 'obsessive*':ti,ab,kw OR 'compulsive*':ti,ab,kw OR 'obsession*':ti,ab,kw OR 'compulsion*':ti,ab,kw OR 'OCD':ti,ab,kw OR 'panic':ti,ab,kw OR ‘panics’:ti,ab,kw OR ‘panicks’:ti,ab,kw OR ‘panicking’:ti,ab,kw OR ‘panicked’:ti,ab,kw OR 'phobi*':ti,ab,kw OR 'catastrophizing*':ti,ab,kw OR 'catastrophising*':ti,ab,kw OR 'catastrophisation*':ti,ab,kw OR 'catastrophization*':ti,ab,kw OR 'catastrophic thinking*':ti,ab,kw OR 'cognitive rumination'/exp OR 'rumination*':ti,ab,kw OR 'ruminative thinking*':ti,ab,kw OR 'psychological distress*':ti,ab,kw OR 'emotional distress*':ti,ab,kw OR 'emotional stress*':ti,ab,kw OR 'distress syndrome*':ti,ab,kw OR 'repetitive behavior*':ti,ab,kw OR 'repetitive behaviour*':ti,ab,kw OR 'mood disorder'/exp OR 'premenstrual syndrome'/exp OR 'depression':ti,ab,kw OR ‘depressions’:ti,ab,kw OR ‘depressive’:ti,ab,kw OR ‘depressed’:ti,ab,kw OR 'unipolar disorder*':ti,ab,kw OR 'MDD':ti,ab,kw OR 'disruptive mood dysregulation disorder*':ti,ab,kw OR 'dysthymi*':ti,ab,kw OR 'disthymi*':ti,ab,kw OR 'dysthimi*':ti,ab,kw OR ‘premenstrual syndrome*’:ti,ab,kw OR 'premenstrual dysphoric disorder*':ti,ab,kw OR 'psychosis'/exp OR 'schizophrenia spectrum disorder'/exp OR 'schizophreni*':ti,ab,kw OR 'schizoaffective*':ti,ab,kw OR 'schizo-affective*':ti,ab,kw OR 'delusional':ti,ab,kw OR 'paranoid':ti,ab,kw OR 'schizotypal':ti,ab,kw OR 'psychotic*':ti,ab,kw OR 'psychosis':ti,ab,kw OR 'psychoses':ti,ab,kw OR 'catatoni*':ti,ab,kw OR 'bipolar*':ti,ab,kw OR 'cyclothymi*':ti,ab,kw OR 'manic':ti,ab,kw OR ‘hypomanic’:ti,ab,kw OR 'mania':ti,ab,kw OR ‘hypomania’:ti,ab,kw OR 'affective disorder*':ti,ab,kw OR 'mood disorder*':ti,ab,kw OR 'disruptive mood dysregulation disorder'/exp OR 'autism'/exp OR 'attention deficit hyperactivity disorder'/exp OR 'autism*':ti,ab,kw OR 'autistic*':ti,ab,kw OR 'ASD':ti,ab,kw OR 'Asperger*':ti,ab,kw OR 'pervasive developmental disorder*':ti,ab,kw OR 'pervasive child developmental disorder*':ti,ab,kw OR 'PDD':ti,ab,kw OR 'attention deficit disorder*':ti,ab,kw OR 'attention deficit hyperactivity disorder*':ti,ab,kw OR 'attention deficit and disruptive behaviour disorder*':ti,ab,kw OR 'attention deficit and disruptive behavior disorder*':ti,ab,kw OR 'ADHD':ti,ab,kw OR 'PDDNOS':ti,ab,kw OR 'PTSD':ti,ab,kw OR 'stress disorder*':ti,ab,kw OR 'acute stress*':ti,ab,kw OR ‘chronic stress*’:ti,ab,kw OR 'reactive attachment disorder*':ti,ab,kw OR 'severe stress*':ti,ab,kw OR 'personality disorder'/exp OR 'personalit*':ti,ab,kw OR 'borderline':ti,ab,kw OR 'antisocial':ti,ab,kw OR 'anti social':ti,ab,kw OR 'histrionic':ti,ab,kw OR 'narcissis*':ti,ab,kw OR ‘narcism’:ti,ab,kw OR ‘narcist*’:ti,ab,kw OR 'avoidant disorder*':ti,ab,kw OR 'schizoid':ti,ab,kw OR ‘sociopath*’:ti,ab,kw OR ‘psychopath*’:ti,ab,kw OR ‘dyssocial’:ti,ab,kw OR ‘dissocial’:ti,ab,kw OR 'social behavior disorder*':ti,ab,kw OR 'social behaviour disorder*':ti,ab,kw OR 'character disorder':ti,ab,kw OR ‘anankastic’:ti,ab,kw OR ‘negativistic’:ti,ab,kw OR ‘sadistic’:ti,ab,kw OR ‘masochistic’:ti,ab,kw OR ‘self defeating’:ti,ab,kw OR 'drug dependence'/exp OR 'drug abuse'/exp OR 'behavioral addiction'/de OR 'computer addiction'/exp OR 'hallucinogen persisting perception disorder'/exp OR 'stimulant use disorder'/exp OR ‘addict*’:ti,ab,kw OR 'pathological gambling'/exp OR ‘substance use*’:ti,ab,kw OR ‘drug use*’:ti,ab,kw OR ‘drug habituation’:ti,ab,kw OR ‘drug bombing’:ti,ab,kw OR ‘drug parachuting’:ti,ab,kw OR ((‘substance’ OR ‘drug’ OR ‘alcohol’ OR ‘solvent’ OR ‘tobacco’) NEXT/1 (‘related’ OR ‘use’ OR ‘induced’) NEXT/1 ‘disorder*’):ti,ab,kw OR ((‘substance’ OR ‘drug’ OR ‘alcohol’ OR ‘solvent’ OR ‘tobacco’ OR ‘internet’ OR ‘videogame’ OR ‘gaming’ OR ‘game’) NEXT/1 (‘dependen*’)):ti,ab,kw OR ((‘substance’ OR ‘drug’ OR ‘alcohol’ OR 'glue' OR ‘solvent’ OR ‘tobacco’) NEXT/1 (‘abuse*’ OR ‘misuse*’)):ti,ab,kw OR 'alcoholism':ti,ab,kw OR ‘alcoholic*’:ti,ab,kw OR ‘alcohol problem*’:ti,ab,kw OR ‘binge drinking’:ti,ab,kw OR ‘binge alcohol consumption’:ti,ab,kw OR ‘heavy drinking’:ti,ab,kw OR 'excessive drinking':ti,ab,kw OR 'hazardous drinking':ti,ab,kw OR 'problem drinking':ti,ab,kw OR 'problematic drinking':ti,ab,kw OR ‘caffeine’:ti,ab,kw OR ‘cannabis’:ti,ab,kw OR ‘marijuana’:ti,ab,kw OR ‘cocaine’:ti,ab,kw OR ‘amphetamine*’:ti,ab,kw OR ‘narcotic*’:ti,ab,kw OR ‘hallucinogen*’:ti,ab,kw OR ‘phencyclidine’:ti,ab,kw OR ‘inhalant*’:ti,ab,kw OR ‘glue sniffing’:ti,ab,kw OR ‘solvent sniffing’:ti,ab,kw OR ‘opioid*’:ti,ab,kw OR ‘opiate*’:ti,ab,kw OR ‘heroin’:ti,ab,kw OR ‘morphine’:ti,ab,kw OR ‘opium’:ti,ab,kw OR ‘anxiolytic*’:ti,ab,kw OR ‘hypnotic*’:ti,ab,kw OR ‘benzodiazepine*’:ti,ab,kw OR ‘sedative*’:ti,ab,kw OR 'tranquilizer*':ti,ab,kw OR 'tranquiliser*':ti,ab,kw OR ‘stimulant*’:ti,ab,kw OR ‘methamphetamine*’:ti,ab,kw OR ‘nicotine’:ti,ab,kw OR ‘gaming disorder*’:ti,ab,kw OR 'compulsive gaming':ti,ab,kw OR ‘compulsive computer gaming’:ti,ab,kw OR ‘compulsive internet gaming’:ti,ab,kw OR ‘compulsive video gaming’:ti,ab,kw OR ‘compulsive videogaming’:ti,ab,kw OR 'pathological gaming':ti,ab,kw OR 'pathological computer gaming':ti,ab,kw OR 'pathological internet gaming':ti,ab,kw OR 'pathological video gaming':ti,ab,kw OR 'pathological videogaming':ti,ab,kw OR 'excessive gaming':ti,ab,kw OR 'excessive computer gaming':ti,ab,kw OR 'excessive internet gaming':ti,ab,kw OR 'excessive video gaming':ti,ab,kw OR 'excessive videogaming':ti,ab,kw OR 'internet use disorder*':ti,ab,kw OR 'internet-related disorder*':ti,ab,kw OR ‘problematic computer use’:ti,ab,kw OR ‘compulsive computer use’:ti,ab,kw OR ‘pathological computer use’:ti,ab,kw OR ‘problematic internet use’:ti,ab,kw OR ‘pathological internet use’:ti,ab,kw OR 'gambling disorder*':ti,ab,kw OR 'pathological gambling':ti,ab,kw OR ‘problem gambling’:ti,ab,kw OR ‘problematic gambling’:ti,ab,kw OR ‘social media disorder*’:ti,ab,kw OR ‘problematic social media use’:ti,ab,kw OR ‘pathological social media use’:ti,ab,kw OR ‘social media use disorder*’:ti,ab,kw OR ‘problematic use of social media’:ti,ab,kw OR ‘problematic screen use’:ti,ab,kw OR ‘problematic mobile phone use’:ti,ab,kw OR ‘problematic smart phone use’:ti,ab,kw OR ‘problematic mobilephone use’:ti,ab,kw OR ‘problematic smartphone use’:ti,ab,kw OR ‘problematic use of mobile phone*’:ti,ab,kw OR ‘problematic use of smart phone*’:ti,ab,kw OR ‘problematic use of mobilephone*’:ti,ab,kw OR ‘problematic use of smartphone*’:ti,ab,kw OR 'severe mental illness*':ti,ab,kw OR 'serious mental illness*':ti,ab,kw OR 'severe mental disorder*':ti,ab,kw OR 'serious mental disorder*':ti,ab,kw) AND ('climate change'/exp OR 'greenhouse effect'/exp OR ‘greenhouse effect’:ti,ab,kw OR 'climate change*':ti,ab,kw OR 'changing climate':ti,ab,kw OR 'climate emergenc*':ti,ab,kw OR 'climate impact*':ti,ab,kw OR 'climate-related impact*':ti,ab,kw OR 'global warming':ti,ab,kw OR 'ecological crisis':ti,ab,kw OR 'ecological threat*':ti,ab,kw OR 'climate crisis':ti,ab,kw OR 'climate disaster*':ti,ab,kw OR 'environmental disaster*':ti,ab,kw OR 'ecological disaster*':ti,ab,kw OR 'ecological loss':ti,ab,kw OR 'ecological problem*':ti,ab,kw OR 'environmental problem*':ti,ab,kw OR 'environmental crisis':ti,ab,kw OR 'environmental change*':ti,ab,kw OR 'environmental threat*':ti,ab,kw OR ‘climate threat*’:ti,ab,kw OR 'environmental destruction*':ti,ab,kw OR 'ecological destruction*':ti,ab,kw OR 'environmental issue*':ti,ab,kw OR 'ecological issue*':ti,ab,kw OR ‘climate issue*’:ti,ab,kw OR 'climate variability':ti,ab,kw OR 'environmental distress*':ti,ab,kw OR 'environmentally-induced distress*':ti,ab,kw OR 'eco-distress*':ti,ab,kw OR 'ecodistress*':ti,ab,kw OR ‘ecological distress*’:ti,ab,kw OR ‘climate distress*’:ti,ab,kw OR ‘climate related distress*’:ti,ab,kw OR 'climate psychology':ti,ab,kw OR 'environmental psychology':ti,ab,kw OR 'eco-psychology':ti,ab,kw OR 'ecopsychology':ti,ab,kw OR 'eco-sickness':ti,ab,kw OR 'ecosickness':ti,ab,kw OR 'eco-anxiety'/exp OR 'eco-anxiety':ti,ab,kw OR 'ecoanxiety':ti,ab,kw OR 'eco-angst':ti,ab,kw OR ‘climate anxiet*’:ti,ab,kw OR ‘climate-related anxiet*’:ti,ab,kw OR ‘environmental anxiet*’:ti,ab,kw OR 'ecophobia':ti,ab,kw OR 'eco-phobia':ti,ab,kw OR 'eco-paralysis':ti,ab,kw OR 'ecoparalysis':ti,ab,kw OR 'eco-fear*':ti,ab,kw OR 'ecofear*':ti,ab,kw OR 'eco-guilt':ti,ab,kw OR 'ecoguilt':ti,ab,kw OR ‘environmental guilt’:ti,ab,kw OR 'eco-sham*':ti,ab,kw OR 'ecosham*':ti,ab,kw OR 'ecological stress*':ti,ab,kw OR 'pre-traumatic stress*':ti,ab,kw OR 'anthropocene disorder*':ti,ab,kw OR 'psychoterratic*':ti,ab,kw OR 'ecological anxiet*':ti,ab,kw OR 'climate guilt':ti,ab,kw OR 'climate-related guilt':ti,ab,kw OR 'climate fear*':ti,ab,kw OR 'climate-related fear*':ti,ab,kw OR 'ecological worr*':ti,ab,kw OR ‘environmental worr*’:ti,ab,kw OR 'ecotrauma*':ti,ab,kw OR 'solastalgia':ti,ab,kw OR 'eco-nostalgia':ti,ab,kw OR 'econostalgia':ti,ab,kw OR 'environmental melanchol*':ti,ab,kw OR 'ecodepression':ti,ab,kw OR 'eco depression':ti,ab,kw OR 'eco-despair':ti,ab,kw OR 'ecodespair':ti,ab,kw OR 'eco-anger':ti,ab,kw OR 'eco-overwhelm':ti,ab,kw OR 'environmentalism'/exp OR ‘environmentalis*’:ti,ab,kw OR ‘environmental advocac*’:ti,ab,kw OR ‘climate advocac*’:ti,ab,kw OR ‘ecological advocac*’:ti,ab,kw OR ‘eco-advocac*’:ti,ab,kw OR 'climate activis*':ti,ab,kw OR ‘environmental activis*’:ti,ab,kw OR ‘ecological activis*’:ti,ab,kw OR ‘eco-activis*’:ti,ab,kw OR ‘pro-environmental behavior’/exp OR ‘pro-environmental behavior*’:ti,ab,kw OR ‘pro-environmental behaviour*’:ti,ab,kw OR ‘proenvironmental behavior*’:ti,ab,kw OR ‘proenvironmental behaviour*’:ti,ab,kw OR ‘ecological behavior’:ti,ab,kw OR ‘ecological behaviour’:ti,ab,kw) NOT ‘conference abstract’:it

**WoS Core Collection (not Book Citation Index Science, Book Citation Index Social Science & Humanities, Index Chemicus en Current Chemical Reactions)**

TS=("posttraumatic stress*" OR "posttraumatic syndrome*" OR "battered child syndrome*" OR "combat disorder*" OR "combat stress disorder*" OR "shell shock*" OR "trauma*" OR "psychotrauma*" OR "emotional damage" OR "emotional harm" OR "emotional injur*" OR "mental damage" OR "mental harm" OR "mental injur*" OR "psychic damage" OR "psychic harm" OR "psychic injur*" OR "psychological damage" OR "psychological harm" OR "psychological injur*" OR "anxiet*" OR "neurotic*" OR "neurosis" OR "neuroses" OR "psychoneurosis" OR "psychoneuroses" OR "psychoneurotic*" OR "obsessive*" OR "compulsive*" OR "obsession*" OR "compulsion*" OR "OCD" OR "panic" OR "panics" OR "panicks" OR "panicking" OR "panicked" OR "phobi*" OR "catastrophizing*" OR "catastrophising*" OR "catastrophisation*" OR "catastrophization*" OR "catastrophic thinking*" OR "rumination*" OR "ruminative thinking*" OR "psychological distress*" OR "emotional distress*" OR "emotional stress*" OR "distress syndrome*" OR "repetitive behavior*" OR "repetitive behaviour*" OR "depression" OR "depressions" OR "depressive" OR "depressed" OR "unipolar disorder*" OR "MDD" OR "disruptive mood dysregulation disorder*" OR "dysthymi*" OR "disthymi*" OR "dysthimi*" OR "premenstrual syndrome*" OR "premenstrual dysphoric disorder*" OR "schizophreni*" OR "schizoaffective*" OR "schizo-affective*" OR "delusional" OR "paranoid" OR "schizotypal" OR "psychotic*" OR "psychosis" OR "psychoses" OR "catatoni*" OR "bipolar*" OR "cyclothymi*" OR "manic" OR "hypomanic" OR "mania" OR "hypomania" OR "affective disorder*" OR "mood disorder*" OR "autism*" OR "autistic*" OR "ASD" OR "Asperger*" OR "pervasive developmental disorder*" OR "pervasive child developmental disorder*" OR "PDD" OR "attention deficit disorder*" OR "attention deficit hyperactivity disorder*" OR "attention deficit and disruptive behaviour disorder*" OR "attention deficit and disruptive behavior disorder*" OR "ADHD" OR "PDDNOS" OR "PTSD" OR "stress disorder*" OR "acute stress*" OR "chronic stress*" OR "reactive attachment disorder*" OR "severe stress*" OR "personalit*" OR "borderline" OR "antisocial" OR "anti social" OR "histrionic" OR "narcissis*" OR "narcism" OR "narcist*" OR "avoidant disorder*" OR "schizoid" OR "sociopath*" OR "psychopath*" OR "dyssocial" OR "dissocial" OR "social behavior disorder*" OR "social behaviour disorder*" OR "character disorder" OR “anankastic” OR "negativistic" OR "sadistic" OR "masochistic" OR "self defeating" OR "addict*" OR "substance use*" OR "drug use*" OR "drug habituation" OR "drug bombing" OR "drug parachuting" OR (("substance" OR "drug" OR "alcohol" OR "solvent" OR "tobacco") NEAR/1 ("related" OR "use" OR "induced") NEAR/1 "disorder*") OR (("substance" OR "drug" OR "alcohol" OR "solvent" OR "tobacco" OR "internet" OR "videogame" OR "gaming" OR "game") NEAR/1 (“dependen*”)) OR (("substance" OR "drug" OR "alcohol" OR "glue" OR "solvent" OR "tobacco") NEAR/1 (“abuse*” OR “misuse*”)) OR "alcoholism" OR "alcoholic*" OR "alcohol problem*" OR "binge drinking" OR "binge alcohol consumption" OR "heavy drinking" OR "excessive drinking" OR "hazardous drinking" OR "problem drinking" OR "problematic drinking" OR "caffeine" OR "cannabis" OR "marijuana" OR "cocaine" OR "amphetamine*" OR "narcotic*" OR "hallucinogen*" OR "phencyclidine" OR "inhalant*" OR "glue sniffing" OR "solvent sniffing" OR "opioid*" OR "opiate*" OR "heroin" OR "morphine" OR "opium" OR "anxiolytic*" OR "hypnotic*" OR "benzodiazepine*" OR "sedative*" OR "tranquilizer*" OR "tranquiliser*" OR "stimulant*" OR "methamphetamine*" OR "nicotine" OR "gaming disorder*" OR "compulsive gaming" OR "compulsive computer gaming" OR "compulsive internet gaming" OR "compulsive video gaming" OR "compulsive videogaming" OR "pathological gaming" OR "pathological computer gaming" OR "pathological internet gaming" OR "pathological video gaming" OR "pathological videogaming" OR "excessive gaming" OR "excessive computer gaming" OR "excessive internet gaming" OR "excessive video gaming" OR "excessive videogaming" OR "internet use disorder*" OR "internet-related disorder*" OR "problematic computer use" OR "compulsive computer use" OR "pathological computer use" OR "problematic internet use" OR "pathological internet use" OR "gambling disorder*" OR "pathological gambling" OR "problem gambling" OR "problematic gambling" OR "social media disorder*" OR "problematic social media use" OR "pathological social media use" OR "social media use disorder*" OR "problematic use of social media" OR "problematic screen use" OR "problematic mobile phone use" OR "problematic smart phone use" OR "problematic mobilephone use" OR "problematic smartphone use" OR "problematic use of mobile phone*" OR "problematic use of smart phone*" OR "problematic use of mobilephone*" OR "problematic use of smartphone*" OR "severe mental illness*" OR "serious mental illness*" OR "severe mental disorder*" OR "serious mental disorder*") AND TS=("greenhouse effect" OR "climate change*" OR "changing climate" OR "climate emergenc*" OR "climate impact*" OR "climate-related impact*" OR "global warming" OR "ecological crisis" OR "ecological threat*" OR "climate crisis" OR "climate disaster*" OR "environmental disaster*" OR "ecological disaster*" OR "ecological loss" OR "ecological problem*" OR "environmental problem*" OR "environmental crisis" OR "environmental change*" OR "environmental threat*" OR "climate threat*" OR "environmental destruction*" OR "ecological destruction*" OR "environmental issue*" OR "ecological issue*" OR "climate issue*" OR "climate variability" OR "environmental distress*" OR "environmentally-induced distress*" OR "eco-distress*" OR "ecodistress*" OR "ecological distress*" OR "climate distress*" OR "climate related distress*" OR "climate psychology" OR "environmental psychology" OR "eco-psychology" OR "ecopsychology" OR "eco-sickness" OR "ecosickness" OR "eco-anxiety" OR "ecoanxiety" OR "eco-angst" OR "climate anxiet*" OR "climate-related anxiet*" OR "environmental anxiet*" OR "ecophobia" OR "eco-phobia" OR "eco-paralysis" OR "ecoparalysis" OR "eco-fear*" OR "ecofear*" OR "eco-guilt" OR "ecoguilt" OR "environmental guilt" OR "eco-sham*" OR "ecosham*" OR "ecological stress*" OR "pre-traumatic stress*" OR "anthropocene disorder*" OR "psychoterratic*" OR "ecological anxiet*" OR "climate guilt" OR "climate-related guilt" OR "climate fear*" OR "climate-related fear*" OR "ecological worr*" OR "environmental worr*" OR "ecotrauma*" OR "solastalgia" OR "eco-nostalgia" OR "econostalgia" OR "environmental melanchol*" OR "ecodepression" OR "eco depression" OR "eco-despair" OR "ecodespair" OR "eco-anger" OR "eco-overwhelm" OR "environmentalis*" OR "environmental advocac*" OR "climate advocac*" OR "ecological advocac*" OR "eco-advocac*" OR "climate activis*" OR "environmental activis*" OR "ecological activis*" OR "eco-activis*" OR "pro-environmental behavior*" OR "pro-environmental behaviour*" OR "proenvironmental behavior*" OR "proenvironmental behaviour*" OR "ecological behavior" OR "ecological behaviour") NOT DT=(“meeting abstract”)

**Scopus**

(TITLE-ABS("posttraumatic stress*" OR "posttraumatic syndrome*" OR "battered child syndrome*" OR "combat disorder*" OR "combat stress disorder*" OR "shell shock*" OR "trauma*" OR "psychotrauma*" OR "emotional damage" OR "emotional harm" OR "emotional injur*" OR "mental damage" OR "mental harm" OR "mental injur*" OR "psychic damage" OR "psychic harm" OR "psychic injur*" OR "psychological damage" OR "psychological harm" OR "psychological injur*" OR "anxiet*" OR "neurotic*" OR "neurosis" OR "neuroses" OR "psychoneurosis" OR "psychoneuroses" OR "psychoneurotic*" OR "obsessive*" OR "compulsive*" OR "obsession*" OR "compulsion*" OR "OCD" OR "panic" OR "panics" OR "panicks" OR "panicking" OR "panicked" OR "phobi*" OR "catastrophizing*" OR "catastrophising*" OR "catastrophisation*" OR "catastrophization*" OR "catastrophic thinking*" OR "rumination*" OR "ruminative thinking*" OR "psychological distress*" OR "emotional distress*" OR "emotional stress*" OR "distress syndrome*" OR "repetitive behavior*" OR "repetitive behaviour*" OR "depression" OR "depressions" OR "depressive" OR "depressed" OR "unipolar disorder*" OR "MDD" OR "disruptive mood dysregulation disorder*" OR "dysthymi*" OR "disthymi*" OR "dysthimi*" OR "premenstrual syndrome*" OR "premenstrual dysphoric disorder*" OR "schizophreni*" OR "schizoaffective*" OR "schizo-affective*" OR "delusional" OR "paranoid" OR "schizotypal" OR "psychotic*" OR "psychosis" OR "psychoses" OR "catatoni*" OR "bipolar*" OR "cyclothymi*" OR "manic" OR "hypomanic" OR "mania" OR "hypomania" OR "affective disorder*" OR "mood disorder*" OR "autism*" OR "autistic*" OR "ASD" OR "Asperger*" OR "pervasive developmental disorder*" OR "pervasive child developmental disorder*" OR "PDD" OR "attention deficit disorder*" OR "attention deficit hyperactivity disorder*" OR "attention deficit and disruptive behaviour disorder*" OR "attention deficit and disruptive behavior disorder*" OR "ADHD" OR "PDDNOS" OR "PTSD" OR "stress disorder*" OR "acute stress*" OR "chronic stress*" OR "reactive attachment disorder*" OR "severe stress*" OR "personalit*" OR "borderline" OR "antisocial" OR "anti social" OR "histrionic" OR "narcissis*" OR "narcism" OR "narcist*" OR "avoidant disorder*" OR "schizoid" OR "sociopath*" OR "psychopath*" OR "dyssocial" OR "dissocial" OR "social behavior disorder*" OR "social behaviour disorder*" OR "character disorder" OR “anankastic” OR "negativistic" OR "sadistic" OR "masochistic" OR "self defeating" OR "addict*" OR "substance use*" OR "drug use*" OR "drug habituation" OR "drug bombing" OR "drug parachuting" OR (("substance" OR "drug" OR "alcohol" OR "solvent" OR "tobacco") W/1 ("related" OR "use" OR "induced") W/1 "disorder*") OR (("substance" OR "drug" OR "alcohol" OR "solvent" OR "tobacco" OR "internet" OR "videogame" OR "gaming" OR "game") W/1 (“dependen*”)) OR (("substance" OR "drug" OR "alcohol" OR "glue" OR "solvent" OR "tobacco") W/1 (“abuse*” OR “misuse*”)) OR "alcoholism" OR "alcoholic*" OR "alcohol problem*" OR "binge drinking" OR "binge alcohol consumption" OR "heavy drinking" OR "excessive drinking" OR "hazardous drinking" OR "problem drinking" OR "problematic drinking" OR "caffeine" OR "cannabis" OR "marijuana" OR "cocaine" OR "amphetamine*" OR "narcotic*" OR "hallucinogen*" OR "phencyclidine" OR "inhalant*" OR "glue sniffing" OR "solvent sniffing" OR "opioid*" OR "opiate*" OR "heroin" OR "morphine" OR "opium" OR "anxiolytic*" OR "hypnotic*" OR "benzodiazepine*" OR "sedative*" OR "tranquilizer*" OR "tranquiliser*" OR "stimulant*" OR "methamphetamine*" OR "nicotine" OR "gaming disorder*" OR "compulsive gaming" OR "compulsive computer gaming" OR "compulsive internet gaming" OR "compulsive video gaming" OR "compulsive videogaming" OR "pathological gaming" OR "pathological computer gaming" OR "pathological internet gaming" OR "pathological video gaming" OR "pathological videogaming" OR "excessive gaming" OR "excessive computer gaming" OR "excessive internet gaming" OR "excessive video gaming" OR "excessive videogaming" OR "internet use disorder*" OR "internet-related disorder*" OR "problematic computer use" OR "compulsive computer use" OR "pathological computer use" OR "problematic internet use" OR "pathological internet use" OR "gambling disorder*" OR "pathological gambling" OR "problem gambling" OR "problematic gambling" OR "social media disorder*" OR "problematic social media use" OR "pathological social media use" OR "social media use disorder*" OR "problematic use of social media" OR "problematic screen use" OR "problematic mobile phone use" OR "problematic smart phone use" OR "problematic mobilephone use" OR "problematic smartphone use" OR "problematic use of mobile phone*" OR "problematic use of smart phone*" OR "problematic use of mobilephone*" OR "problematic use of smartphone*" OR "severe mental illness*" OR "serious mental illness*" OR "severe mental disorder*" OR "serious mental disorder*") OR AUTHKEY("posttraumatic stress*" OR "posttraumatic syndrome*" OR "battered child syndrome*" OR "combat disorder*" OR "combat stress disorder*" OR "shell shock*" OR "trauma*" OR "psychotrauma*" OR "emotional damage" OR "emotional harm" OR "emotional injur*" OR "mental damage" OR "mental harm" OR "mental injur*" OR "psychic damage" OR "psychic harm" OR "psychic injur*" OR "psychological damage" OR "psychological harm" OR "psychological injur*" OR "anxiet*" OR "neurotic*" OR "neurosis" OR "neuroses" OR "psychoneurosis" OR "psychoneuroses" OR "psychoneurotic*" OR "obsessive*" OR "compulsive*" OR "obsession*" OR "compulsion*" OR "OCD" OR "panic" OR "panics" OR "panicks" OR "panicking" OR "panicked" OR "phobi*" OR "catastrophizing*" OR "catastrophising*" OR "catastrophisation*" OR "catastrophization*" OR "catastrophic thinking*" OR "rumination*" OR "ruminative thinking*" OR "psychological distress*" OR "emotional distress*" OR "emotional stress*" OR "distress syndrome*" OR "repetitive behavior*" OR "repetitive behaviour*" OR "depression" OR "depressions" OR "depressive" OR "depressed" OR "unipolar disorder*" OR "MDD" OR "disruptive mood dysregulation disorder*" OR "dysthymi*" OR "disthymi*" OR "dysthimi*" OR "premenstrual syndrome*" OR "premenstrual dysphoric disorder*" OR "schizophreni*" OR "schizoaffective*" OR "schizo-affective*" OR "delusional" OR "paranoid" OR "schizotypal" OR "psychotic*" OR "psychosis" OR "psychoses" OR "catatoni*" OR "bipolar*" OR "cyclothymi*" OR "manic" OR "hypomanic" OR "mania" OR "hypomania" OR "affective disorder*" OR "mood disorder*" OR "autism*" OR "autistic*" OR "ASD" OR "Asperger*" OR "pervasive developmental disorder*" OR "pervasive child developmental disorder*" OR "PDD" OR "attention deficit disorder*" OR "attention deficit hyperactivity disorder*" OR "attention deficit and disruptive behaviour disorder*" OR "attention deficit and disruptive behavior disorder*" OR "ADHD" OR "PDDNOS" OR "PTSD" OR "stress disorder*" OR "acute stress*" OR "chronic stress*" OR "reactive attachment disorder*" OR "severe stress*" OR "personalit*" OR "borderline" OR "antisocial" OR "anti social" OR "histrionic" OR "narcissis*" OR "narcism" OR "narcist*" OR "avoidant disorder*" OR "schizoid" OR "sociopath*" OR "psychopath*" OR "dyssocial" OR "dissocial" OR "social behavior disorder*" OR "social behaviour disorder*" OR "character disorder" OR “anankastic” OR "negativistic" OR "sadistic" OR "masochistic" OR "self defeating" OR "addict*" OR "substance use*" OR "drug use*" OR "drug habituation" OR "drug bombing" OR "drug parachuting" OR (("substance" OR "drug" OR "alcohol" OR "solvent" OR "tobacco") W/1 ("related" OR "use" OR "induced") W/1 "disorder*") OR (("substance" OR "drug" OR "alcohol" OR "solvent" OR "tobacco" OR "internet" OR "videogame" OR "gaming" OR "game") W/1 (“dependen*”)) OR (("substance" OR "drug" OR "alcohol" OR "glue" OR "solvent" OR "tobacco") W/1 (“abuse*” OR “misuse*”)) OR "alcoholism" OR "alcoholic*" OR "alcohol problem*" OR "binge drinking" OR "binge alcohol consumption" OR "heavy drinking" OR "excessive drinking" OR "hazardous drinking" OR "problem drinking" OR "problematic drinking" OR "caffeine" OR "cannabis" OR "marijuana" OR "cocaine" OR "amphetamine*" OR "narcotic*" OR "hallucinogen*" OR "phencyclidine" OR "inhalant*" OR "glue sniffing" OR "solvent sniffing" OR "opioid*" OR "opiate*" OR "heroin" OR "morphine" OR "opium" OR "anxiolytic*" OR "hypnotic*" OR "benzodiazepine*" OR "sedative*" OR "tranquilizer*" OR "tranquiliser*" OR "stimulant*" OR "methamphetamine*" OR "nicotine" OR "gaming disorder*" OR "compulsive gaming" OR "compulsive computer gaming" OR "compulsive internet gaming" OR "compulsive video gaming" OR "compulsive videogaming" OR "pathological gaming" OR "pathological computer gaming" OR "pathological internet gaming" OR "pathological video gaming" OR "pathological videogaming" OR "excessive gaming" OR "excessive computer gaming" OR "excessive internet gaming" OR "excessive video gaming" OR "excessive videogaming" OR "internet use disorder*" OR "internet-related disorder*" OR "problematic computer use" OR "compulsive computer use" OR "pathological computer use" OR "problematic internet use" OR "pathological internet use" OR "gambling disorder*" OR "pathological gambling" OR "problem gambling" OR "problematic gambling" OR "social media disorder*" OR "problematic social media use" OR "pathological social media use" OR "social media use disorder*" OR "problematic use of social media" OR "problematic screen use" OR "problematic mobile phone use" OR "problematic smart phone use" OR "problematic mobilephone use" OR "problematic smartphone use" OR "problematic use of mobile phone*" OR "problematic use of smart phone*" OR "problematic use of mobilephone*" OR "problematic use of smartphone*" OR "severe mental illness*" OR "serious mental illness*" OR "severe mental disorder*" OR "serious mental disorder*")) AND (TITLE-ABS("greenhouse effect" OR "climate change*" OR "changing climate" OR "climate emergenc*" OR "climate impact*" OR "climate-related impact*" OR "global warming" OR "ecological crisis" OR "ecological threat*" OR "climate crisis" OR "climate disaster*" OR "environmental disaster*" OR "ecological disaster*" OR "ecological loss" OR "ecological problem*" OR "environmental problem*" OR "environmental crisis" OR "environmental change*" OR "environmental threat*" OR "climate threat*" OR "environmental destruction*" OR "ecological destruction*" OR "environmental issue*" OR "ecological issue*" OR "climate issue*" OR "climate variability" OR "environmental distress*" OR "environmentally-induced distress*" OR "eco-distress*" OR "ecodistress*" OR "ecological distress*" OR "climate distress*" OR "climate related distress*" OR "climate psychology" OR "environmental psychology" OR "eco-psychology" OR "ecopsychology" OR "eco-sickness" OR "ecosickness" OR "eco-anxiety" OR "ecoanxiety" OR "eco-angst" OR "climate anxiet*" OR "climate-related anxiet*" OR "environmental anxiet*" OR "ecophobia" OR "eco-phobia" OR "eco-paralysis" OR "ecoparalysis" OR "eco-fear*" OR "ecofear*" OR "eco-guilt" OR "ecoguilt" OR "environmental guilt" OR "eco-sham*" OR "ecosham*" OR "ecological stress*" OR "pre-traumatic stress*" OR "anthropocene disorder*" OR "psychoterratic*" OR "ecological anxiet*" OR "climate guilt" OR "climate-related guilt" OR "climate fear*" OR "climate-related fear*" OR "ecological worr*" OR "environmental worr*" OR "ecotrauma*" OR "solastalgia" OR "eco-nostalgia" OR "econostalgia" OR "environmental melanchol*" OR "ecodepression" OR "eco depression" OR "eco-despair" OR "ecodespair" OR "eco-anger" OR "eco-overwhelm" OR "environmentalis*" OR "environmental advocac*" OR "climate advocac*" OR "ecological advocac*" OR "eco-advocac*" OR "climate activis*" OR "environmental activis*" OR "ecological activis*" OR "eco-activis*" OR "pro-environmental behavior*" OR "pro-environmental behaviour*" OR "proenvironmental behavior*" OR "proenvironmental behaviour*" OR "ecological behavior" OR "ecological behaviour") OR AUTHKEY("greenhouse effect" OR "climate change*" OR "changing climate" OR "climate emergenc*" OR "climate impact*" OR "climate-related impact*" OR "global warming" OR "ecological crisis" OR "ecological threat*" OR "climate crisis" OR "climate disaster*" OR "environmental disaster*" OR "ecological disaster*" OR "ecological loss" OR "ecological problem*" OR "environmental problem*" OR "environmental crisis" OR "environmental change*" OR "environmental threat*" OR "climate threat*" OR "environmental destruction*" OR "ecological destruction*" OR "environmental issue*" OR "ecological issue*" OR "climate issue*" OR "climate variability" OR "environmental distress*" OR "environmentally-induced distress*" OR "eco-distress*" OR "ecodistress*" OR "ecological distress*" OR "climate distress*" OR "climate related distress*" OR "climate psychology" OR "environmental psychology" OR "eco-psychology" OR "ecopsychology" OR "eco-sickness" OR "ecosickness" OR "eco-anxiety" OR "ecoanxiety" OR "eco-angst" OR "climate anxiet*" OR "climate-related anxiet*" OR "environmental anxiet*" OR "ecophobia" OR "eco-phobia" OR "eco-paralysis" OR "ecoparalysis" OR "eco-fear*" OR "ecofear*" OR "eco-guilt" OR "ecoguilt" OR "environmental guilt" OR "eco-sham*" OR "ecosham*" OR "ecological stress*" OR "pre-traumatic stress*" OR "anthropocene disorder*" OR "psychoterratic*" OR "ecological anxiet*" OR "climate guilt" OR "climate-related guilt" OR "climate fear*" OR "climate-related fear*" OR "ecological worr*" OR "environmental worr*" OR "ecotrauma*" OR "solastalgia" OR "eco-nostalgia" OR "econostalgia" OR "environmental melanchol*" OR "ecodepression" OR "eco depression" OR "eco-despair" OR "ecodespair" OR "eco-anger" OR "eco-overwhelm" OR "environmentalis*" OR "environmental advocac*" OR "climate advocac*" OR "ecological advocac*" OR "eco-advocac*" OR "climate activis*" OR "environmental activis*" OR "ecological activis*" OR "eco-activis*" OR "pro-environmental behavior*" OR "pro-environmental behaviour*" OR "proenvironmental behavior*" OR "proenvironmental behaviour*" OR "ecological behavior" OR "ecological behaviour"))

**Cochrane**

([mh ^"Anxiety Disorders"] OR [mh "Neurotic Disorders"] OR [mh ^"Obsessive-Compulsive Disorder"] OR [mh ^"Obsessive Behavior"] OR [mh "Panic Disorder"] OR [mh ^"Phobic Disorders"] OR [mh "Psychological Distress"] OR [mh ^"Catastrophization"] OR (anxiet* OR neurotic* OR "neurosis" OR "neuroses" OR "psychoneurosis" OR "psychoneuroses" OR psychoneurotic* OR obsessive* OR compulsive* OR obsession* OR compulsion* OR "OCD" OR "panic" OR "panics" OR "panicks" OR "panicking" OR "panicked" OR phobi* OR catastrophizing* OR catastrophising* OR catastrophisation* OR catastrophization* OR (catastrophic NEXT thinking*)):ti,ab,kw OR [mh "Rumination, Cognitive"] OR (rumination* OR (ruminative NEXT thinking*) OR (psychological NEXT distress*) OR (emotional NEXT distress*) OR (emotional NEXT stress*) OR (distress NEXT syndrome*) OR (repetitive NEXT behavior*) OR (repetitive NEXT behaviour*)):ti,ab,kw OR [mh "Mood Disorders"] OR [mh "Depression"] OR [mh "Premenstrual Syndrome"] OR ("depression" OR "depressions" OR "depressive" OR "depressed" OR (unipolar NEXT disorder*) OR "MDD" OR (disruptive NEXT mood NEXT dysregulation NEXT disorder*) OR dysthymi* OR disthymi* OR dysthimi* OR (premenstrual NEXT syndrome*) OR (premenstrual NEXT dysphoric NEXT disorder*)):ti,ab,kw OR [mh "Schizophrenia Spectrum and Other Psychotic Disorders"] OR (schizophreni* OR schizoaffective* OR schizo-affective* OR "delusional" OR "paranoid" OR "schizotypal" OR psychotic* OR "psychosis" OR "psychoses" OR catatoni* OR bipolar* OR cyclothymi*):ti,ab,kw OR [mh "Mania"] OR ("manic" OR "hypomanic" OR hypomania* OR "mania" OR (affective NEXT disorder*) OR (mood NEXT disorder*)):ti,ab,kw OR [mh "Autism Spectrum Disorder"] OR [mh "Attention Deficit Disorder with Hyperactivity"] OR (autism* OR autistic* OR Asperger* OR (pervasive NEXT developmental NEXT disorder*) OR (pervasive NEXT child NEXT developmental NEXT disorder*) OR "PDD" OR "ASD" OR (attention NEXT deficit NEXT disorder*) OR (attention NEXT deficit NEXT hyperactivity NEXT disorder*) OR ("attention deficit and disruptive" NEXT behaviour NEXT disorder*) OR ("attention deficit and disruptive" NEXT behavior NEXT disorder*) OR "ADHD" OR "PDDNOS"):ti,ab,kw OR [mh "Stress Disorders, Traumatic"] OR (trauma* OR psychotrauma* OR "emotional damage" OR "emotional harm" OR (emotional NEXT injur*) OR "mental damage" OR "mental harm" OR (mental NEXT injur*) OR "psychic damage" OR "psychic harm" OR (psychic NEXT injur*) OR "psychological damage" OR "psychological harm" OR (psychological NEXT injur*) OR (battered NEXT child NEXT syndrome*) OR (combat NEXT disorder*) OR (combat NEXT stress NEXT disorder*) OR (shell NEXT shock*) OR "PTSD" OR (posttraumatic NEXT stress*) OR (posttraumatic NEXT syndrome*) OR (stress NEXT disorder*) OR (acute NEXT stress*) OR (chronic NEXT stress*) OR (reactive NEXT attachment NEXT disorder*) OR (severe NEXT stress*)):ti,ab,kw OR [mh "Personality Disorders"] OR (personalit* OR "borderline" OR "antisocial" OR "anti social" OR "histrionic" OR narcissis* OR "narcism" OR narcist* OR (avoidant NEXT disorder*) OR "schizoid" OR sociopath* OR psychopath* OR dyssocial OR dissocial OR (social NEXT behavior NEXT disorder*) OR (social NEXT behaviour NEXT disorder*) OR (character NEXT disorder*) OR "anankastic" OR "negativistic" OR "sadistic" OR "masochistic" OR "self defeating"):ti,ab,kw OR [mh "Substance-Related Disorders"] OR [mh ^"Behavior, Addictive"] OR (addict*):ti,ab,kw OR [mh "Technology Addiction"] OR [mh "Gambling"] OR ((substance NEXT related NEXT disorder*) OR (substance NEXT abuse*) OR (drug NEXT abuse*) OR (drug NEXT misuse*) OR (substance NEXT use*) OR (drug NEXT use*) OR "drug habituation" OR "drug bombing" OR "drug parachuting" OR (substance NEXT induced NEXT disorder*) OR (substance NEXT dependen*) OR (drug NEXT related NEXT disorder*) OR (drug NEXT induced NEXT disorder*) OR (drug NEXT dependen*) OR (alcohol NEXT related NEXT disorder*) OR (alcohol NEXT use NEXT disorder*) OR (alcohol NEXT induced NEXT disorder*) OR "alcoholism" OR alcoholic* OR (alcohol NEXT problem*) OR (alcohol NEXT abuse*) OR (alcohol NEXT dependen*) OR "binge drinking" OR "binge alcohol consumption" OR "heavy drinking" OR "excessive drinking" OR "hazardous drinking" OR "problem drinking" OR "problematic drinking" OR "caffeine" OR "cannabis" OR "marijuana" OR "cocaine" OR amphetamine* OR narcotic* OR hallucinogen* OR "phencyclidine" OR inhalant* OR (glue NEXT abuse*) OR "glue sniffing" OR (solvent NEXT abuse*) OR "solvent sniffing" OR (solvent NEXT related NEXT disorder*) OR (solvent NEXT dependen*) OR (solvent NEXT use NEXT disorder*) OR (solvent NEXT induced NEXT disorder*) OR opioid* OR opiate* OR "heroin" OR "morphine" OR "opium" OR anxiolytic* OR hypnotic* OR benzodiazepine* OR sedative* OR tranquilizer* OR tranquiliser* OR stimulant* OR methamphetamine* OR (tobacco NEXT related NEXT disorder*) OR (tobacco NEXT use NEXT disorder*) OR (tobacco NEXT induced NEXT disorder*) OR (tobacco NEXT dependen*) OR (tobacco NEXT abuse*) OR "nicotine" OR (gaming NEXT disorder*) OR (game NEXT dependen*) OR (videogame NEXT dependen*) OR (gaming NEXT dependen*) OR "compulsive gaming" OR "compulsive computer gaming" OR "compulsive internet gaming" OR "compulsive video gaming" OR "compulsive videogaming" OR "pathological gaming" OR "pathological computer gaming" OR "pathological internet gaming" OR "pathological video gaming" OR "pathological videogaming" OR "excessive gaming" OR "excessive computer gaming" OR "excessive internet gaming" OR "excessive video gaming" OR "excessive videogaming" OR (internet NEXT use NEXT disorder*) OR ("internet-related" NEXT disorder*) OR "problematic computer use" OR "pathological computer use" OR "problematic internet use" OR "pathological internet use" OR (internet NEXT dependen*) OR (gambling NEXT disorder*) OR "pathological gambling" OR "problem gambling" OR "problematic gambling" OR (social NEXT media NEXT disorder*) OR "problematic social media use" OR "pathological social media use" OR (social NEXT media NEXT use NEXT disorder*) OR "problematic use of social media" OR "problematic screen use" OR "problematic mobile phone use" OR "problematic smart phone use" OR "problematic mobilephone use" OR "problematic smartphone use" OR ("problematic use of" NEXT mobile NEXT phone*) OR ("problematic use of" NEXT smart NEXT phone*) OR ("problematic use of" NEXT mobilephone*) OR ("problematic use of" NEXT smartphone*) OR (severe NEXT mental NEXT illness*) OR (serious NEXT mental NEXT illness*) OR (severe NEXT mental NEXT disorder*) OR (serious NEXT mental NEXT disorder*)):ti,ab,kw) AND ([mh "Climate Change"] OR [mh "Greenhouse Effect"] OR ("greenhouse effect" OR (climate NEXT change*) OR "changing climate" OR (climate NEXT emergenc*) OR (climate NEXT impact*) OR ("climate-related" NEXT impact*) OR "global warming" OR "ecological crisis" OR (ecological NEXT threat*) OR "climate crisis" OR (climate NEXT disaster*) OR (environmental NEXT disaster*) OR (ecological NEXT disaster*) OR "ecological loss" OR (ecological NEXT problem*) OR (environmental NEXT problem*) OR "environmental crisis" OR (environmental NEXT change*) OR (environmental NEXT threat*) OR (climate NEXT threat*) OR (environmental NEXT destruction*) OR (ecological NEXT destruction*) OR (environmental NEXT issue*) OR (ecological NEXT issue*) OR (climate NEXT issue*) OR "climate variability" OR (environmental NEXT distress*) OR "environmentally-induced distress" OR (eco NEXT distress*) OR ecodistress* OR (ecological NEXT distress*) OR (climate NEXT distress*) OR ("climate-related" NEXT distress*) OR "climate psychology" OR "environmental psychology" OR "eco-psychology" OR "ecopsychology" OR "eco-sickness" OR "ecosickness" OR "eco-anxiety" OR "ecoanxiety" OR "eco-angst" OR (climate NEXT anxiet*) OR "climate-related anxiety" OR (environmental NEXT anxiet*) OR "ecophobia" OR "eco-phobia" OR "eco-paralysis" OR "ecoparalysis" OR (eco NEXT fear*) OR ecofear* OR "eco-guilt" OR "ecoguilt" OR "environmental guilt" OR (eco NEXT sham*) OR ecosham* OR (ecological NEXT stress*) OR ("pre-traumatic" NEXT stress*) OR (Anthropocene NEXT disorder*) OR psychoterratic* OR "ecological anxiety" OR "climate guilt" OR "climate-related guilt" OR (climate NEXT fear*) OR ("climate-related" NEXT fear*) OR (ecological NEXT worr*) OR (environmental NEXT worr*) OR ecotrauma* OR "solastalgia" OR "eco-nostalgia" OR "econostalgia" OR (environmental NEXT melanchol*) OR "eco-depression" OR "ecodepression" OR "eco-despair" OR "ecodespair" OR "eco-anger" OR "eco-overwhelm"):ti,ab,kw OR [mh "Environmentalism"] OR (environmentalis* OR (environmental NEXT advocac*) OR (climate NEXT advocac*) OR (ecological NEXT advocac*) OR (eco NEXT advocac*) OR (climate NEXT activis*) OR (environmental NEXT activis*) OR (ecological NEXT activis*) OR (eco NEXT activis*) OR ("pro-environmental" NEXT behavior*) OR ("pro-environmental" NEXT behaviour*) OR (proenvironmental NEXT behavior*) OR (proenvironmental NEXT behaviour*) OR (ecological NEXT behavior*) OR (ecological NEXT behaviour*)):ti,ab,kw)
